# Harnessing plant lignin for sustainable materials and chemicals: integrating biosynthesis, structural diversity, and circular bioeconomy perspectives

**DOI:** 10.3389/fpls.2026.1730538

**Published:** 2026-03-04

**Authors:** Haixin Jiao, Rania Al-Tohamy, Mohammed Hussein M. Alsharbaty, Jianzhong Sun, Min Xiong, Michael Schagerl, Sameh S. Ali

**Affiliations:** 1 School of Environmental Science and Engineering, Yancheng Institute of Technology, Yancheng, China; 2 Biofuels Institute, School of the Environment and Safety Engineering, Jiangsu University, Zhenjiang, China; 3 Branch of Prosthodontics, College of Dentistry, University of Al-Ameed, Karbala, Iraq; 4 Department of Functional and Evolutionary Ecology, University of Vienna, Vienna, Austria; 5 Botany and Microbiology Department, Faculty of Science, Tanta University, Tanta, Egypt

**Keywords:** bioplastics, biopolymers, carbon fibers, circular bioeconomy, lignin valorization, lignin-based nanocomposites, plant biomass utilization

## Abstract

Lignin is the most abundant renewable source of aromatic carbon on Earth and a central yet historically underutilized component of lignocellulosic biomass. Its complex and heterogeneous molecular architecture has long constrained efficient and selective conversion into value-added products, despite its high aromatic carbon content and chemical functionality. Recent advances in lignin extraction, fractionation, modification, and application-driven design have substantially expanded the range of achievable material and chemical performance within circular bioeconomy frameworks. This review provides a comprehensive and critically integrated assessment of lignin valorization that explicitly links plant biosynthesis and structural diversity to industrial convertibility, functional materials development, and sustainability performance. Green extraction technologies—including deep eutectic solvent and hydrotropic systems—are evaluated with respect to lignin structural quality, energy demand, solvent recovery, and downstream compatibility. Targeted chemical and enzymatic modification strategies enabling more reproducible lignin streams are discussed alongside applications in carbon fibers, nanomaterials, adhesives, bioplastics, cementitious systems, and additive manufacturing. Quantitative benchmarking against fossil-based incumbents identifies application domains where lignin-derived materials already achieve comparable performance, as well as areas where intrinsic structural limitations remain. In parallel, catalytic depolymerization pathways toward renewable aromatic chemicals are assessed from both mechanistic and systems-level perspectives. Environmental and economic implications are critically examined using recent life-cycle and techno-economic evidence, highlighting the influence of allocation choices, energy integration, and comparison with lignin incineration for energy recovery. Overall, this review clarifies how application-targeted lignin design and system-level sustainability assessment are essential for translating lignin’s biological complexity into scalable, competitive solutions for sustainable materials and chemicals.

## 1 Introduction

**Figure 1:**
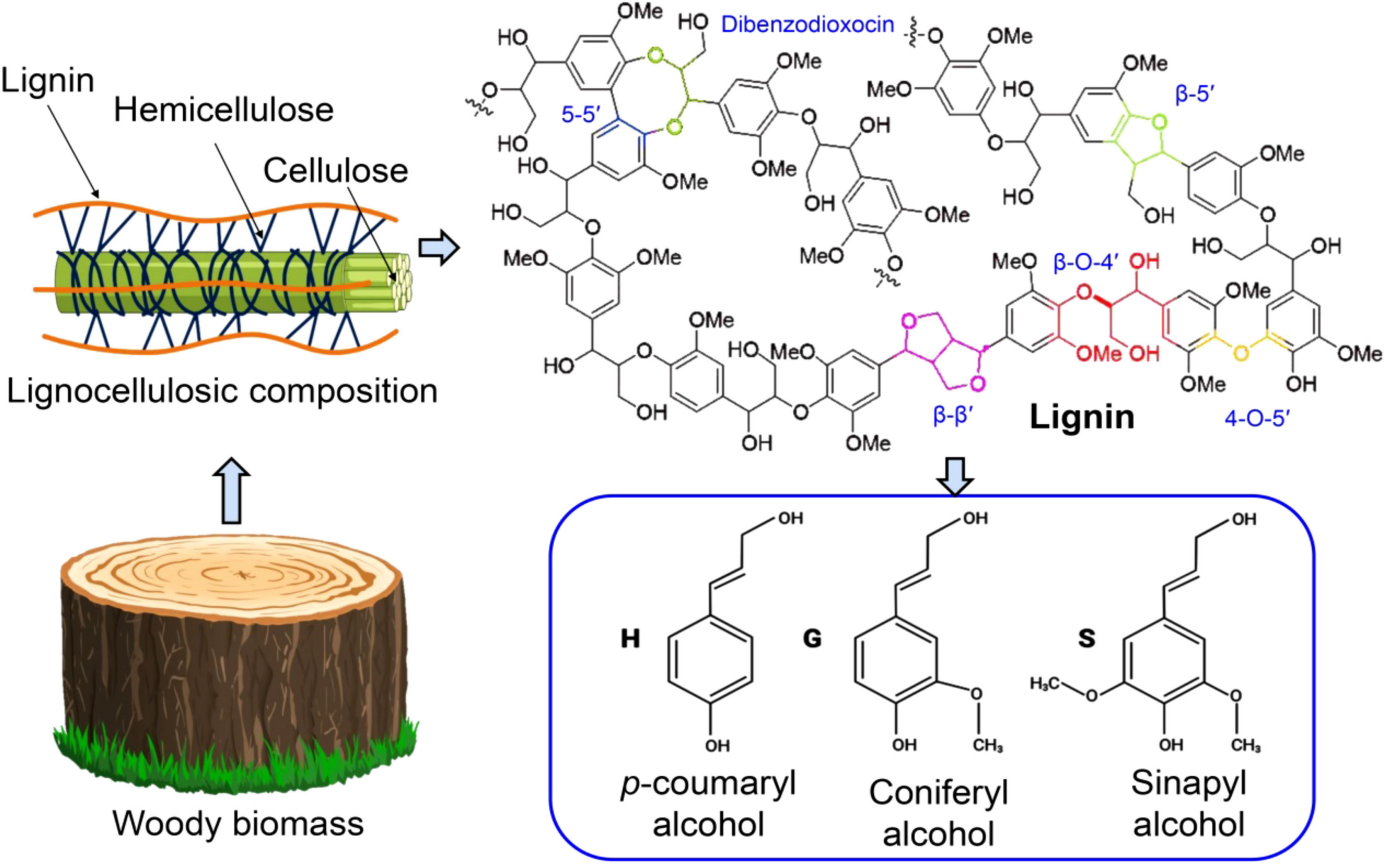
Schematic representation of lignocellulosic biomass and lignin structural heterogeneity. Woody biomass is composed of cellulose microfibrils embedded in a matrix of hemicellulose and lignin. Lignin is formed from the oxidative coupling of p-coumaryl (H), coniferyl (G), and sinapyl (S) alcohols, generating a heterogeneous polymer network connected by a variety of ether (e.g., β–O–4′) and carbon–carbon (e.g., β–β′, β–5′, 5–5′) linkages.

Lignin is a highly abundant, renewable aromatic biopolymer that provides structural rigidity, hydrophobicity, and defense to plant secondary cell walls, particularly in woody and vascular tissues (Moradian et al., 2026). It is widely recognized as the second most abundant natural polymer after cellulose and constitutes a substantial fraction of lignocellulosic biomass. Recent plant-biology and biorefining studies emphasize that lignin’s essential biological function is also the primary origin of its resistance to deconstruction and its strong influence on biomass conversion efficiency (Dixon et al., 2024; Arasu et al., 2025). As illustrated in **Figure 1**, lignin is embedded within the lignocellulosic matrix alongside cellulose and hemicellulose and is composed of randomly coupled *p*-hydroxyphenyl (H), guaiacyl (G), and syringyl (S) units linked through diverse ether and carbon–carbon bonds, highlighting the structural heterogeneity that underpins both its biological role and processing challenges.

Chemically, lignin is formed via oxidative coupling of phenylpropanoid-derived monolignols, generating a polymer that is inherently heterogeneous rather than a single well-defined macromolecule (Ali et al., 2026). Its linkage distribution, S/G/H composition, functional group density, molecular-weight distribution, and degree of branching vary with plant species, tissue type, growth conditions, and processing history. This structural variability is consistently identified as a primary reason why universal or “one-size-fits-all” processing strategies are rarely effective (Sethupathy et al., 2022; Daelemans et al., 2025). At the same time, lignin’s aromatic backbone and oxygen-rich functional groups make it the only large-volume renewable source of aromatics for materials and chemicals, sustaining strong interest in its utilization within circular bioeconomy frameworks (Dixon et al., 2024).

From an industrial perspective, the principal limitation of lignin valorization is not complexity alone, but the impact of heterogeneity on reactivity control, processability, and batch-to-batch reproducibility. Technical lignins produced at scale—particularly kraft lignin—are structurally “process-made,” as pulping conditions induce cleavage and condensation reactions that increase resistant C–C linkages and alter functional group profiles (Ali et al., 2024). These changes complicate solubility control and downstream functionalization, hindering resin synthesis and polymer design when consistent properties are required (Daelemans et al., 2025). Broad molecular-weight distributions and variable thermal transitions further destabilize melt-processing windows, which is especially critical for lignin-based carbon materials where precursor uniformity strongly affects final performance (Sethupathy et al., 2022). In addition, compositional heterogeneity arising from residual carbohydrates, ash, sulfur, and metal ions can promote side reactions and catalyst deactivation in chemical upgrading routes, reducing yields and complicating scale-up (Argyropoulos et al., 2023). These constraints explain why heterogeneity reduction is increasingly framed as a prerequisite for reliable lignin product manufacturing rather than a secondary optimization step (Ali et al., 2021a; Daelemans et al., 2025).

Despite these challenges, lignin’s aromaticity, high carbon content, and multifunctionality support a wide range of valorization pathways spanning renewable chemicals and advanced materials. Selective depolymerization strategies aim to generate bio-based aromatics capable of displacing fossil-derived phenols and related intermediates, although product distributions and selectivity remain highly sensitive to lignin structure and isolation history (Daelemans et al., 2025). In parallel, lignin can function as a macromolecular building block or functional additive in resins, composites, coatings, and carbon materials; however, these applications require controlled interfacial behavior and predictable structure–property relationships, again emphasizing the need for lignin streams with reduced heterogeneity and well-defined specifications (Argyropoulos et al., 2023).

Accordingly, substantial recent progress has focused on extraction, fractionation, and modification strategies that improve lignin quality and reproducibility while reducing environmental impact. Beyond conventional kraft and organosolv isolation, greener solvent platforms such as deep eutectic solvents (DES) and hydrotropic systems have been actively developed to enable delignification under milder or more selective conditions and to better preserve structurally useful motifs, while accounting for solvent recovery, mass transfer, and energy demand (Ji et al., 2022; Li et al., 2023; Abolore et al., 2025). In parallel, post-isolation fractionation approaches have been increasingly applied to narrow molecular-weight distributions and reduce chemical diversity, directly addressing heterogeneity-related limitations in processing and performance (Liu et al., 2021). These upstream quality-engineering strategies are now widely viewed as enabling technologies for both materials- and chemicals-oriented lignin valorization pathways (Abolore et al., 2025; Daelemans et al., 2025). At the same time, sustainability claims for lignin valorization increasingly require evidence beyond feedstock renewability, particularly because lignin is often combusted for on-site heat and power in pulp mills. Recent life-cycle assessment (LCA) studies emphasize that environmental outcomes depend strongly on allocation choices (including the zero-burden assumption), energy mix, solvent recovery, and the counterfactual baseline of lignin incineration for energy recovery, reinforcing that sustainability must be treated as a system-level property rather than assumed *a priori* (Kylili et al., 2023; Staccioli et al., 2024).

In this context, the objective of this review is to provide an integrated and critical assessment of lignin valorization that explicitly connects plant-derived structural diversity to industrial convertibility and circular bioeconomy implementation. The review focuses on (i) biosynthetic and structural determinants governing lignin heterogeneity and reactivity, (ii) recent advances in green extraction and fractionation technologies that enable reproducible, application-ready lignin streams, (iii) targeted modification strategies that enhance solubility, compatibility, and functional performance, and (iv) application-driven valorization pathways spanning materials, composites, and catalytic depolymerization to renewable aromatics. In addition, environmental and economic implications are critically evaluated using recent life-cycle and techno-economic evidence, with emphasis on system boundaries, allocation choices, and comparison with lignin incineration for energy recovery. By integrating molecular structure, processing strategies, applications, and sustainability assessment, this review aims to provide a coherent framework for guiding future lignin research and industrial deployment.

## 2 Molecular architecture and functional diversity of lignin

Lignin is one of the most abundant and structurally intricate biopolymers in nature, serving as a cornerstone of plant architecture and a key determinant of adaptation to terrestrial environments (Jalal et al., 2025). Its complexity originates from a highly heterogeneous and irregular three-dimensional network formed through the oxidative coupling of phenylpropanoid precursors. Within the plant cell wall, lignin provides rigidity, hydrophobicity, and resistance to microbial and enzymatic degradation, thereby enabling efficient water transport, mechanical support, and long-term structural stability. At the biospheric scale, lignin contributes significantly to carbon sequestration, biomass persistence, and ecosystem resilience, making it a central subject in plant biology, biogeochemistry, and sustainable bioeconomy research (Fazeli et al., 2024).

At the molecular level, lignin is a polyphenolic macromolecule composed primarily of three monolignols—H, G, and S units—biosynthesized through the phenylpropanoid pathway. The relative abundance and spatial distribution of these monomers vary widely across plant species, tissues, and developmental stages, resulting in distinct lignin chemotypes with different reactivities and physicochemical properties (Letourneau and Volmer, 2023). Hardwood lignins are typically enriched in S-units, which introduce more ether linkages and greater conformational flexibility, whereas softwood lignins are dominated by G-units that promote extensive crosslinking and increased recalcitrance (Kozhevnikov et al., 2024). This compositional diversity reflects a highly regulated biosynthetic system that allows plants to optimize cell wall performance, hydraulic conductivity, and defense responses under diverse environmental conditions.

Beyond monomer composition, lignin exhibits remarkable functional group diversity, including phenolic and aliphatic hydroxyls, benzyl alcohols, carbonyls, and methoxyl groups (Shu et al., 2021). These functionalities govern lignin’s interactions with polysaccharides, proteins, and inorganic components within the cell wall, influencing supramolecular organization and crosslinking patterns. They also underpin lignin’s antioxidant, UV-absorbing, and antimicrobial properties, which play critical roles in protecting plant tissues against oxidative stress, radiation damage, and pathogen invasion. Consequently, lignin-rich tissues such as xylem, bark, and seed coats exhibit exceptional mechanical robustness and resistance to degradation (Balk et al., 2023).

**Figure 2:**
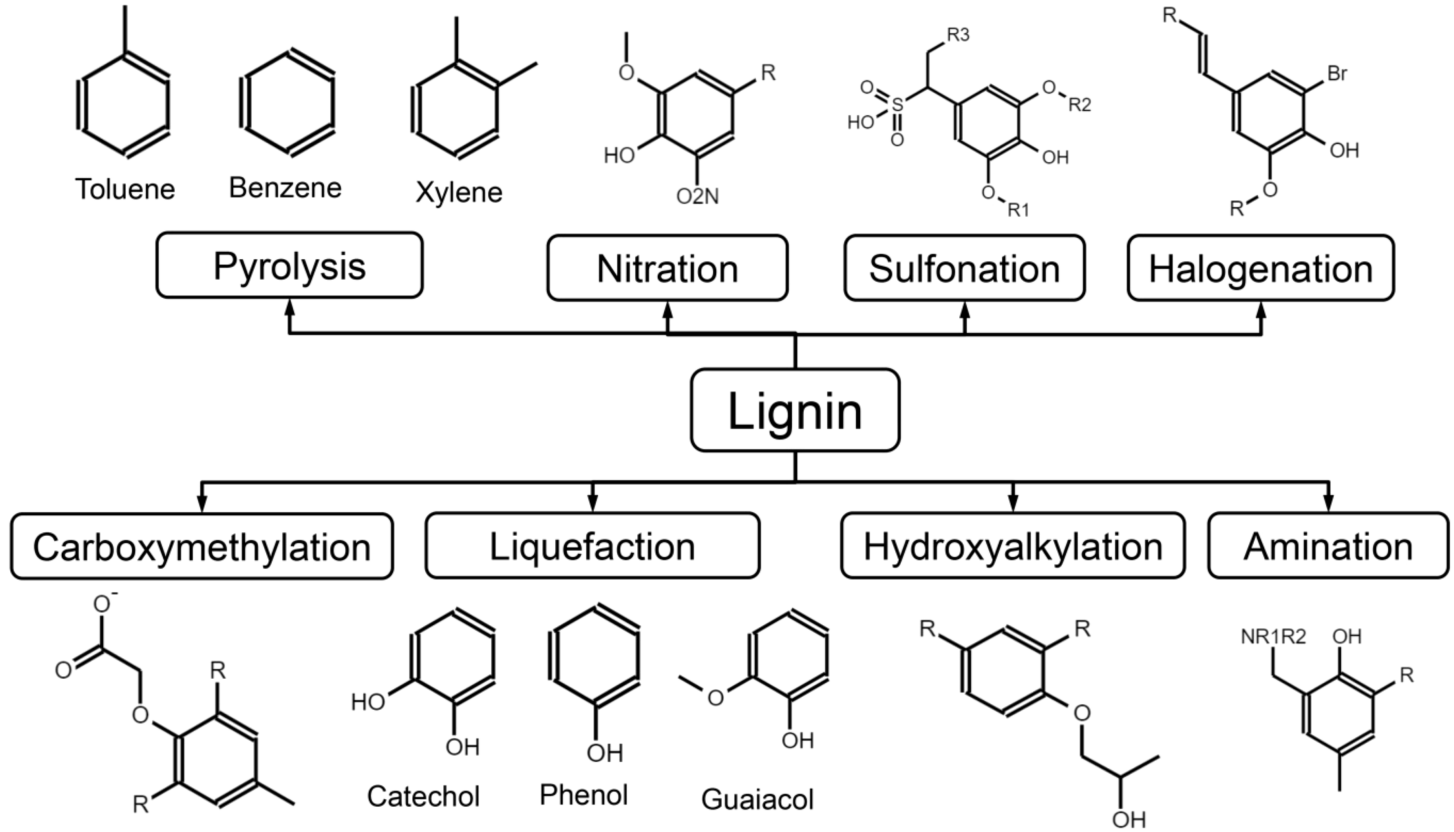
Schematic overview of representative lignin transformation pathways toward value-added aromatic compounds and functional derivatives.

While lignin’s molecular complexity is biologically advantageous, it also dictates the extent to which lignin can be transformed into value-added products, thereby linking plant biochemistry to renewable resource utilization. Through chemical or thermochemical conversion, lignin can be depolymerized into smaller aromatic compounds such as guaiacol, catechol, and phenol, which serve as precursors for resins, antioxidants, flavoring agents, and specialty chemicals (Pandey and Kim, 2011; Ali et al., 2021b). **Figure 2** summarizes the principal transformation pathways of lignin, illustrating how both depolymerization routes (e.g., liquefaction and pyrolysis) and targeted chemical functionalization reactions (such as sulfonation, hydroxyalkylation, amination, nitration, and halogenation) generate a spectrum of aromatic intermediates and functional derivatives relevant to materials and chemical applications. Liquefaction typically yields phenolic intermediates, whereas pyrolysis generates biochar alongside volatile aromatics including benzene, toluene, and xylene (Nadányi et al., 2022). These pathways mirror, to some extent, the natural degradation processes occurring during plant litter decomposition and soil carbon cycling.

Despite this versatility, lignin utilization remains constrained by its heterogeneous structure and inconsistent reactivity, which complicate extraction, modification, and product standardization (Jiju et al., 2025). Irregular interunit linkages, variable monomer ratios, and strong intermolecular associations hinder predictable processing and limit uniform functionalization. Consequently, a detailed understanding of lignin structure—from molecular motifs to supramolecular organization—is essential for designing controlled transformation strategies. From a plant science perspective, such knowledge also provides the foundation for engineering biomass with tailored lignin architectures that balance mechanical function with enhanced degradability or processability (Jiang et al., 2023).

Lignin’s structural heterogeneity further reflects its adaptive and evolutionary significance. Plants actively modulate lignin content and composition in response to environmental stresses such as drought, ultraviolet radiation, and pathogen attack, highlighting lignin’s central role in resilience and resource-use efficiency. Although this variability poses challenges for industrial utilization, it represents a biological advantage that enables fine-tuning of wall mechanics and defense capacity (Kantaros et al., 2024). Advances in enzymatic and genetic engineering have demonstrated that lignin’s aromatic framework can be selectively modified through targeted interventions in the phenylpropanoid pathway, opening opportunities for precision lignin design in crops and trees. The rapid development of high-resolution analytical techniques—including multidimensional nuclear magnetic resonance, mass spectrometry, and synchrotron-based imaging—combined with emerging computational and machine-learning tools, is accelerating the characterization and prediction of lignin structure–function relationships (Beaucamp et al., 2022).

The structural complexity and diversity of lignin are therefore central to both its biological significance and its technological promise (Zhao et al., 2025). Its multifaceted molecular architecture enables plants to thrive under diverse environmental conditions while simultaneously providing a renewable reservoir of aromatic carbon. A deeper comprehension of lignin’s structure, biosynthetic regulation, and reactivity is pivotal for bridging plant science with sustainable innovation and circular bioeconomy strategies (Vasile and Baican, 2023).

From a materials and valorization perspective, a central long-term objective—or “holy grail”—of lignin biosynthesis research is the rational engineering of lignin structure to simultaneously satisfy plant performance requirements and downstream processing efficiency. Rather than minimizing lignin content outright, contemporary strategies aim to redesign lignin architecture to enhance depolymerizability, extractability, and functional uniformity without compromising plant growth, mechanical integrity, or stress resistance (Ralph et al., 2004). Key targets include increasing the abundance of cleavable β–O–4′ ether linkages, tuning the S/G ratio to reduce condensation propensity, and incorporating chemically labile motifs—often referred to as “zip-lignin”—that facilitate selective bond cleavage during chemical or thermal processing (Wilkerson et al., 2014; Vanholme et al., 2019). From a materials perspective, additional biosynthetic goals include narrowing lignin molecular weight distribution, controlling functional group density, and improving structural consistency across biomass sources to enable predictable performance in polymers, carbon materials, and composite systems (Ragauskas et al., 2014; Presley et al., 2021). Ultimately, the transformative potential of lignin lies not in eliminating its recalcitrance, but in aligning plant-evolved functionality with industrial convertibility, thereby creating biomass feedstocks inherently suited for sustainable materials and chemical production.

## 3 Lignin valorization within the circular bioeconomy bridges plant metabolism with sustainable industrial innovation

Lignin has emerged as a pivotal renewable resource within the circular bioeconomy, where the sustainable use of biological carbon seeks to replace fossil-derived feedstocks and minimize waste through closed material cycles. Owing to its abundance, aromatic backbone, and structural versatility, lignin provides a unique link between plant metabolism and the production of renewable materials and chemicals. Within this framework, lignin valorization enables the transformation of plant-fixed carbon into functional products, improving overall biomass utilization efficiency while reducing reliance on non-renewable resources (Singh et al., 2023).

From an environmental perspective, lignin-based products can contribute to climate change mitigation by displacing fossil-derived fuels, polymers, and chemicals with alternatives derived from photosynthetically fixed carbon (Wu et al., 2025a). Unlike petrochemical materials, lignin originates from biomass participating in the short-term carbon cycle, offering the potential for reduced life-cycle greenhouse gas emissions when appropriately integrated into biorefinery systems. In addition, lignin’s inherent aromaticity and functional group diversity support the development of materials and chemicals aligned with green chemistry principles, including reduced toxicity and improved environmental compatibility (Jiju et al., 2025). These attributes position lignin as a strategic component in the transition toward decarbonized industrial processes.

The integration of lignin valorization into modern biorefineries represents a shift from linear biomass processing toward circular, multi-product utilization schemes (John et al., 2025). Historically, lignin generated during pulp or bioethanol production has been primarily combusted on-site for energy recovery, reflecting both its perceived low value and the challenges associated with its conversion. However, lignin’s complex aromatic structure also enables access to higher-value products such as resins, polymeric materials, and carbon-based functional materials, which can significantly enhance the economic performance of lignocellulosic biorefineries (Bass and Epps, 2021). Valorizing lignin alongside cellulose and hemicellulose therefore supports integrated systems in which fuels, materials, and chemicals are co-produced from a single renewable feedstock, consistent with circular bioeconomy principles. Beyond environmental and technical considerations, lignin valorization also carries socioeconomic implications. The development of lignin-based value chains can create new markets for agricultural and forestry residues, support decentralized processing infrastructures, and strengthen regional bioeconomies (Trezza et al., 2025). Such systems have the potential to promote rural development, generate employment, and enhance the resilience of biomass-based industries, although these benefits remain context-dependent (Lizundia et al., 2021).

Despite its promise, the effective incorporation of lignin into circular bioeconomy systems remains constrained by challenges associated with its heterogeneous structure and variable reactivity. Lignin composition and molecular architecture depend strongly on plant species, developmental stage, and environmental conditions, while industrial extraction further modifies its structure and functionality (Balk et al., 2023; Jalal et al., 2025). This variability complicates process standardization and limits predictability in downstream conversion. Addressing these challenges requires coordinated advances across plant biology, chemistry, and process engineering, including targeted manipulation of lignin biosynthesis, improved extraction and fractionation strategies, and the development of selective catalytic, enzymatic, or biological conversion routes. Ultimately, lignin’s role within the circular bioeconomy depends on the ability to translate its biological complexity into controlled, application-ready material streams. Efficient extraction, fractionation, and modification strategies that preserve or tailor lignin’s functional features are therefore foundational to unlocking its value across materials and chemicals applications. These enabling technologies form the basis for reliable lignin valorization and are examined in detail in the following section.

## 4 Lignin extraction and structural modification strategies

In the context of a circular bioeconomy, lignin extraction and modification strategies must simultaneously address efficiency, environmental compatibility, and the preservation of molecular features required for downstream valorization. As a structurally complex polyphenolic macromolecule embedded within the plant secondary cell wall, lignin presents a dual challenge: it is inherently recalcitrant due to its covalent association with polysaccharides, yet it is chemically rich and highly attractive as a renewable aromatic resource. Effective extraction is therefore a prerequisite for transforming lignin into functional biomaterials and value-added chemicals. Contemporary strategies increasingly move beyond simple delignification yield as a performance metric, emphasizing instead lignin quality, structural integrity, and compatibility with targeted applications (Terzopoulou et al., 2025). While conventional alkaline and acid-based processes such as kraft and sulfite pulping remain industrially dominant (Jahan et al., 2021), their tendency to induce condensation, sulfur incorporation, and structural modification limits lignin’s suitability for advanced material applications. Consequently, greener and more selective extraction technologies—combined with post-extraction modification—have emerged as enabling steps in repositioning lignin from an energy by-product to a strategic feedstock for sustainable materials innovation (Fan et al., 2022).

### 4.1 Advances in lignin extraction techniques

Recent advances in lignin extraction reflect a clear shift from severity-driven delignification toward quality-driven recovery, where the objective is to isolate lignin in a form suitable for predictable downstream processing. Modern extraction strategies span mechanical, physicochemical, chemical, enzymatic, and solvent-based approaches, each addressing different aspects of lignin accessibility, selectivity, and structural preservation (Dandash et al., 2025). Rather than representing competing alternatives, these methods increasingly function as complementary tools within integrated biorefinery schemes, where extraction chemistry must be aligned with feedstock characteristics and intended valorization routes.

#### 4.1.1 Mechanical and physicochemical approaches

Mechanical and physicochemical methods primarily serve as preparatory or enabling steps that improve biomass accessibility and enhance the effectiveness of subsequent chemical or solvent-based lignin extraction. Size-reduction techniques such as milling increase surface area and disrupt cell wall architecture, facilitating solvent penetration and mass transfer. Recent developments emphasize the integration of mechanical treatments with thermal or solvent-assisted processes to improve extraction efficiency while limiting uncontrolled lignin degradation. Hybrid techniques such as ultrasonic- and microwave-assisted extraction have demonstrated the ability to enhance lignin yield and purity by selectively disrupting lignocellulosic structures under relatively mild conditions (Zou et al., 2024). For example, Jang et al. (2020) extracted lignin from *Miscanthus* using a combination of ball milling, hydrothermal treatment, and dioxane-based solvents. This integrated approach increased lignin and total phenolic yields while preserving high-molecular-weight lignin fractions through careful control of milling duration and solvent composition. These studies highlight that mechanical and physicochemical treatments are most effective when applied as part of a broader extraction strategy, where they reduce structural barriers without becoming the primary source of lignin chemical modification.

#### 4.1.2 Green chemical and enzymatic strategies for lignin extraction

Chemical and enzymatic extraction strategies represent a transitional space between conventional pulping and fully solvent-driven green technologies, offering improved selectivity while retaining operational familiarity. Organosolv processes, in particular, have gained attention as cleaner alternatives to kraft or sulfite pulping, employing organic solvents—often with mild acid catalysis—to solubilize lignin while limiting condensation reactions and carbohydrate degradation (Tian et al., 2017). Khongchamnan et al. (2021) demonstrated that careful optimization of solvent composition and reaction parameters enables high lignin recovery and cellulose retention, underscoring the importance of process tuning rather than severity.

Enzymatic approaches further enhance selectivity by exploiting the bond-specific catalytic activity of enzymes such as laccases and peroxidases. These systems operate under aqueous, low-temperature conditions and selectively cleave lignin–carbohydrate or phenolic linkages, minimizing nonspecific degradation (Asim et al., 2020). Studies by Gutiérrez et al. (2012) and Hämäläinen et al. (2018) showed that enzymatic delignification can reduce lignin molecular weight and improve solubility without significantly compromising polysaccharide integrity. While enzymatic methods face challenges related to reaction time and enzyme cost, they play an increasingly important role in hybrid extraction schemes where chemical and biological selectivity are combined to balance efficiency and lignin quality. Representative examples of these integrated approaches are summarized in **Table 1**.

**Table 1:** Representative methods for lignin extraction from lignocellulosic biomass.

Biomass source	Extraction method	Key reaction conditions	Lignin yield/characteristics	Key outcomes	Reference
Sieved lignocellulosic biomass	Aldehyde-stabilized lignin extraction	Aldehyde stabilization (6–7 h); drying at 60 °C (16 h)	~40–50% of Klason lignin	Produces stabilized, less-condensed lignin with high monomer yields upon depolymerization; enables efficient cellulose and hemicellulose recovery	Talebi Amiri et al. (2019)
Lignocellulosic waste	Bio-based solvent system (levulinic acid/formic acid)	Temperature, solvent ratio, and time optimized	Yield dependent on temperature and time	Higher temperature and longer extraction increase yield but reduce lignin purity; optimized conditions balance yield and quality	Magalhães et al. (2021)
Marine residues, agri-food byproducts, forest residues, perennial grasses	Microwave-assisted DES pretreatment	800 W, 60 s microwave irradiation	High purity lignin (ChCl–oxalic acid DES)	Rapid and energy-efficient fractionation; trade-off between lignin purity and yield depending on DES composition	Kammoun et al. (2021)
Lignocellulosic biomass	Laccase-mediated delignification	Batch reactor; enzymatic treatment	~40% delignification	Enhances enzymatic hydrolysis (1.4× glucose yield); phenolic inhibitors require recovery for optimal saccharification	Chavan and Gaikwad (2021)
Lignocellulosic biomass	Chemical, physical, mechanical, and enzymatic treatments	Method-dependent	Technical lignins with varied structures	Extraction route strongly influences lignin molecular weight, functionality, and suitability for biopolymers and chemicals	Xu and Ferdosian (2017)
Invasive weed biomass	1,4-dioxane solvent extraction	Acid pretreatment followed by solvent extraction	8.6–11.1 wt% lignin; T_50_ ≈ 490 °C	Lignin exhibits high thermal stability; solvent recovery of 75–79%	Borah et al. (2023)
Lignocellulosic biomass	Protic ionic liquid extraction	100 °C, 2 h	Up to 73% lignin yield	High lignin and sugar recovery under mild conditions; effective delignification and saccharification	Asim et al. (2020)
Lignocellulosic biomass	Hydrothermal pretreatment + solvent extraction + wet milling	190–220 °C pretreatment; enzymatic hydrolysis (6–72 h)	Surface-extracted lignin	Surface lignin removal and wet milling increase cellulose digestibility to ≥95%	Nitsos et al. (2019)
Eucalyptus wood fibers	scCO_2_–ethanol/water extraction	180 °C; TG analysis (30–800 °C)	High-purity lignin with higher S-unit content	scCO_2_-extracted lignin shows superior UV resistance and mechanical reinforcement in composite films	Feng et al. (2022)
Softwood and hardwood biomass	Sub-/supercritical fluid extraction (SFE, CXLE)	60 °C, 350 bar, 30 min	Solvent-dependent yields	Ethyl lactate and acetone provide highest lignin yields; rapid and green extraction	Nardella et al. (2023)
Palm fiber	Ethanol extraction optimized by response surface methodology	174 °C, 111 min	56.2% lignin yield; 91.8% solvent recovery	Extracted lignin successfully converted into activated carbon fibers with enhanced performance	Fan et al. (2022)

DES, deep eutectic solvent; ChCl, choline chloride; LA, levulinic acid; FA, formic acid; scCO_2_, supercritical carbon dioxide; SFE, supercritical fluid extraction; CXLE, CO_2_-expanded liquid extraction; TG, thermogravimetric; T_50_, temperature at 50% weight loss.

#### 4.1.3 Deep eutectic solvent-based extraction of lignin

**Figure 3:**
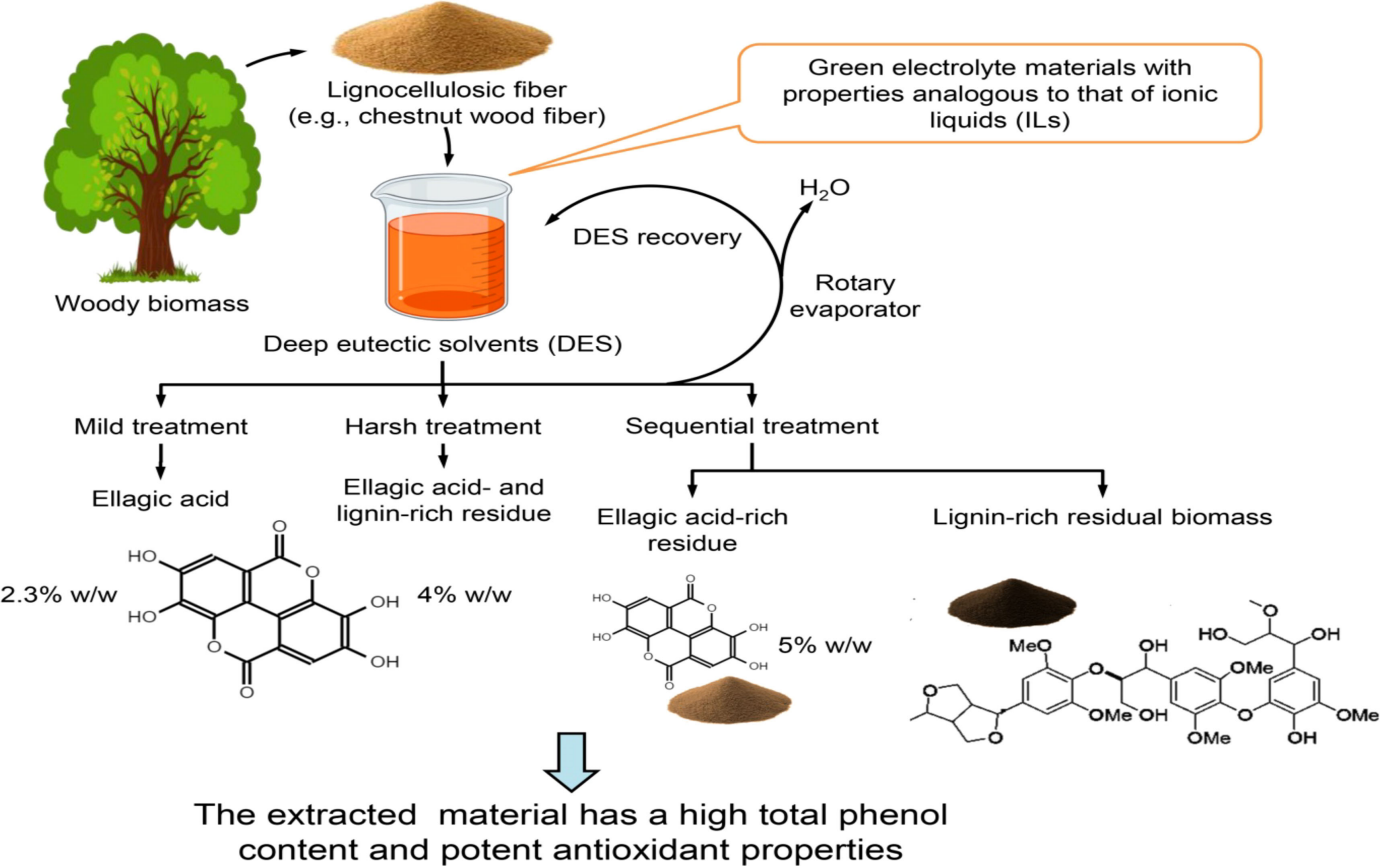
Schematic illustration of deep eutectic solvent (DES)-based extraction and fractionation of lignocellulosic biomass.

DES have recently emerged as one of the most versatile and environmentally benign media for lignin extraction, representing a major advance in sustainable biorefinery technology. DES are a new class of green solvents formed by the combination of a hydrogen-bond donor and a hydrogen-bond acceptor, producing a eutectic mixture with a melting point significantly lower than that of its individual components (Hou et al., 2018). Functionally, DES behave similarly to ionic liquids but are far less expensive, biodegradable, and easier to prepare. Their exceptional ability to dissolve lignin under relatively mild temperatures and pressures makes them highly suitable for processing lignocellulosic biomass, while their recyclability and low volatility align with the principles of green chemistry (Tian et al., 2017). As illustrated in **Figure 3**, deep eutectic solvent systems enable tunable extraction pathways under mild to sequential treatment conditions, allowing selective recovery of phenolic-rich fractions and lignin-enriched residues while maintaining solvent recyclability. In this system, lignocellulosic biomass such as chestnut wood fiber is treated with DES composed of naturally derived components, resulting in the selective recovery of lignin and phenolic compounds such as ellagic acid. The DES can be recovered and reused via a simple evaporation step, ensuring minimal solvent loss and waste generation. The extracted products exhibit high total phenol content and strong antioxidant activity, underscoring the dual benefit of lignin valorization and co-extraction of bioactive compounds.

A representative study by Moccia et al. (2022) demonstrated the efficiency of this approach using a two-step DES treatment of chestnut wood fiber. In the first stage, choline chloride combined with tartaric acid served as the solvent system, resulting in a significant yield of ellagic acid (2.3–4% w/w), surpassing previously reported DES-based extractions from agricultural residues. A subsequent treatment of the residual biomass with choline chloride and lactic acid enabled the isolation of a structurally uniform guaiacyl–syringyl lignin, characterized by high purity and minimal condensation. Both extract fractions showed elevated total phenolic content and strong antioxidant capacity, confirming the ability of DES systems to recover functional lignin while simultaneously generating high-value co-products (Wang et al., 2025).

The adaptability of DES formulations—achieved through adjusting the molar ratio and chemical identity of donor and acceptor components—allows fine control over lignin solubility, selectivity, and structural preservation. Moreover, the mild operating conditions (moderate temperature and near-neutral pH) help retain lignin’s reactive functional groups, making the extracted material suitable for polymer synthesis, coatings, and antioxidant additives. The successful application of DES extraction thus represents a decisive step toward the development of integrated, eco-efficient lignin recovery systems, bridging fundamental plant polymer chemistry with industrial sustainability.

#### 4.1.4 Hydrotropic extraction for high-purity lignin recovery

**Figure 4:**
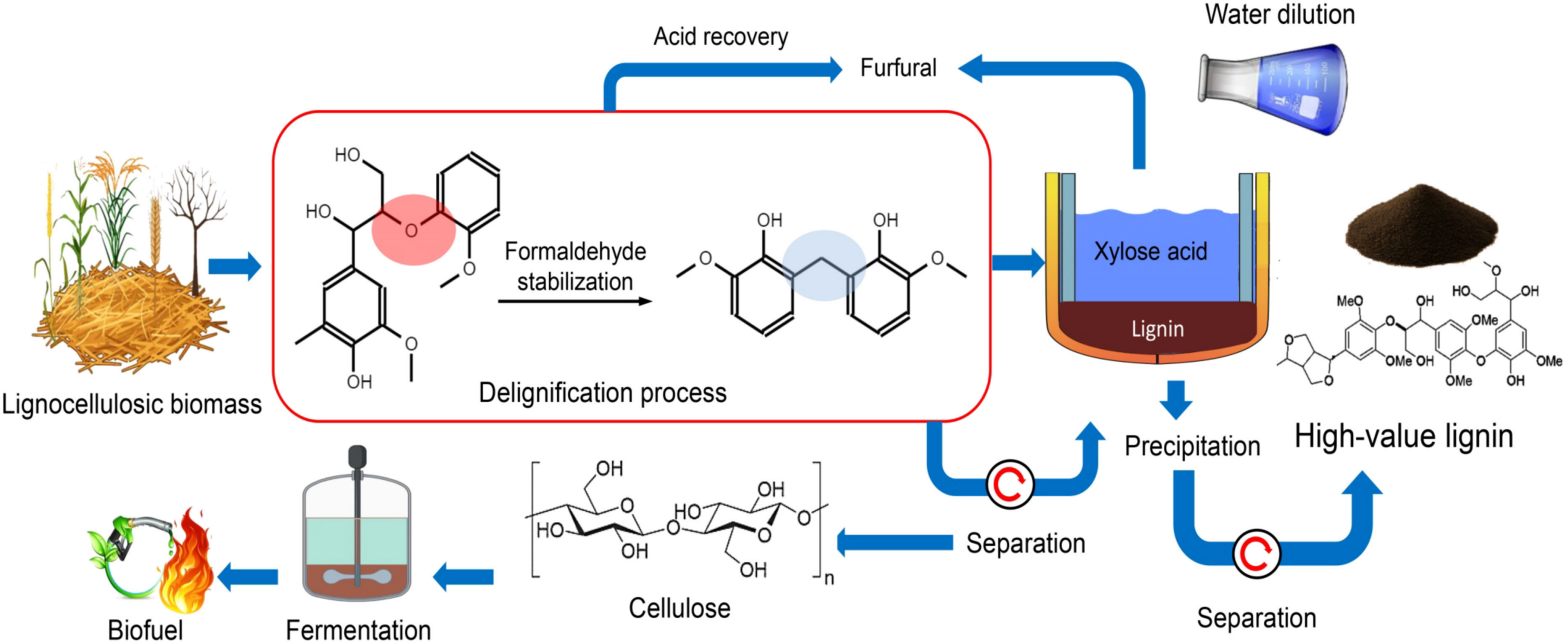
Schematic representation of an integrated lignocellulosic biomass fractionation and stabilization process.

Hydrotropic extraction represents one of the most recent and refined innovations in lignin isolation, offering an environmentally compatible and highly selective approach to recover lignin from lignocellulosic biomass. This technique employs hydrotropes—small, water-soluble organic molecules capable of enhancing the solubility of hydrophobic compounds in aqueous systems—to dissolve lignin without relying on harsh chemical agents. As illustrated in **Figure 4**, stabilization-assisted delignification strategies can suppress lignin condensation during extraction, enabling the recovery of structurally preserved, high-value lignin alongside carbohydrate streams within an integrated biorefinery framework. Hydrotropes function by forming dynamic molecular aggregates that disrupt the lignin network and promote solubilization, thereby facilitating extraction while preserving the structural integrity of other wall components such as cellulose and hemicellulose (Tanis et al., 2024).

Recent research has substantially improved the selectivity and efficiency of hydrotropic extraction systems, making them competitive with conventional alkaline and organosolv processes. Olsson et al. (2019) demonstrated that hydrotropic agents can effectively solubilize lignin from lignocellulosic matrices while leaving cellulose and hemicellulose largely intact. Similarly, Wu et al. (2020) reported that the process can be conducted under relatively mild operational conditions—typically at neutral pH and temperatures below 100 °C—thereby preserving lignin’s native aromatic structure and reactive functional groups. Such mild conditions not only reduce energy demand but also prevent secondary condensation reactions that would otherwise diminish the quality and reactivity of the recovered lignin. The resulting material exhibits high purity and structural uniformity, making it an attractive feedstock for the synthesis of value-added chemicals, resins, and polymeric materials (Jiang et al., 2023).

Further optimization of hydrotropic systems has revealed their strong potential for scalable biorefinery applications. Chen et al. (2017) achieved 90% lignin removal using 80 wt% p-toluenesulfonic acid (p-TsOH) at 80 °C for 20 minutes—a level of efficiency comparable to conventional alkaline extraction that requires significantly higher temperatures (≈150 °C) and extended reaction times exceeding 10 hours. This high performance under mild conditions demonstrates the ability of hydrotropic agents to rapidly delignify diverse biomass sources such as rice straw, sugarcane bagasse, and bamboo. Moreover, the process allows for straightforward acid recovery and reuse, aligning with the principles of resource circularity and waste minimization (**Figure 4**).

The comparative performance of hydrotropic extraction alongside other lignin recovery approaches is summarized in **Table 2**. Mechanical and physicochemical techniques, while simple, often lack selectivity and produce lignin of lower quality. Chemical and enzymatic extraction methods can achieve higher purity but are limited by reagent costs and environmental burdens. DES systems offer excellent tunability and green solvent recovery, whereas hydrotropic systems provide a unique balance of efficiency, scalability, and environmental safety (Suthar, 2025). The optimal choice of extraction route thus depends on desired product characteristics, feedstock type, and processing economics. Overall, the field of lignin extraction is evolving toward more sustainable and integrated biorefinery models. Hydrotropic extraction, in particular, exemplifies this transformation by combining operational simplicity, recyclability, and selectivity within a low-impact framework. The advances in hydrotropic chemistry, together with mechanical, enzymatic, and DES-based innovations, are progressively converting lignin from an industrial by-product into a renewable and multifunctional bioresource (Tanis et al., 2024). Beyond isolation, post-extraction modification serves as the key step that transforms lignin from a recalcitrant structural biopolymer into a sustainable functional material, bridging plant biochemistry with industrial innovation.

**Table 2:** Advantages and limitations of lignin extraction techniques.

Extraction method	Advantages	Limitations	References
Mechanical and physicochemical	Solvent-free or low-chemical approaches; environmentally benign; suitable as pretreatment methods	Energy-intensive; limited selectivity; lignin yield and purity strongly depend on biomass type and operating conditions	Tribot et al. (2019); Nitsos et al. (2019)
Chemical (acid, alkali, organosolv)	Effective disruption of lignocellulosic structure; enables high lignin yields and relatively high purity	Generates chemical waste streams; requires neutralization, solvent recovery, and corrosion-resistant equipment	Gao et al. (2013); Wang et al. (2019)
Enzymatic	High selectivity; preserves lignin structure for high-value applications; reduced chemical inputs	Long processing times; enzyme cost and limited scalability remain major barriers	Gao et al. (2013)
Deep eutectic solvents (DES)	Low volatility and toxicity; tunable solvent systems; effective lignin solubilization while enabling downstream functionalization	Performance depends on DES composition and feedstock; solvent recovery and recyclability remain challenges	Chen et al. (2020)
Hydrotropic extraction	Operates under mild conditions; high lignin purity; good selectivity toward lignin over carbohydrates	Requires large solvent volumes; solvent recovery can be energy-intensive; economic feasibility depends on solvent recycling efficiency	Tian et al. (2017)

#### 4.1.5 Comparative trade-offs between deep eutectic solvent and hydrotropic lignin extraction

DES systems and hydrotropic extraction have emerged as leading green alternatives to conventional lignin isolation technologies; however, they rely on fundamentally different solubilization mechanisms and therefore exhibit distinct advantages and limitations depending on the intended lignin valorization pathway. A meaningful comparison between these two approaches requires evaluation beyond delignification yield alone, incorporating lignin structural quality, solvent recovery strategy, and the distribution of energy demand across reaction and separation steps. Recent studies emphasize that sustainable lignin extraction must be assessed at the process level rather than based solely on operating temperature or reaction severity (Chen et al., 2017; Abolore et al., 2025).

DES-based extraction is generally preferable when lignin is intended for downstream material applications that require controlled molecular architecture and preserved functional group chemistry. DES systems dissolve lignin primarily through hydrogen-bonding interactions and polarity matching, and their chemical composition can be tuned by selecting appropriate hydrogen bond donors and acceptors. This tunability allows selective solubilization while limiting excessive condensation reactions, resulting in lignin fractions with higher phenolic hydroxyl content and narrower molecular weight distributions, which are advantageous for polymer synthesis, coatings, and composite materials (Li et al., 2023). Several studies have demonstrated that DES-extracted lignin exhibits improved reactivity and compatibility with polymeric matrices compared with lignin obtained from harsher chemical treatments, supporting its use in high-value material systems rather than bulk fuel or energy recovery.

Hydrotropic extraction, in contrast, is generally favored when rapid and extensive delignification is required under mild thermal conditions and when process throughput is a dominant consideration. Acid hydrotropes such as p-TsOH enhance lignin solubility by forming transient molecular aggregates in aqueous media, enabling near-complete lignin dissolution at temperatures below 80 °C and residence times on the order of minutes (Chen et al., 2017; Yin et al., 2021). This unusually fast delignification kinetics distinguishes hydrotropic systems from DES and many conventional pulping methods, making them attractive for high-throughput biorefinery operations. Lignin recovery is typically achieved by dilution below the minimum hydrotrope concentration, which induces spontaneous precipitation without additional reagents, simplifying the isolation step (Wang et al., 2020).

Despite these advantages, the trade-offs between yield, purity, and energy demand differ substantially between the two extraction strategies. Hydrotropic extraction often achieves high delignification efficiency, but this does not necessarily translate into optimal lignin recovery yield or structural quality. The acidic environment and high hydrotrope concentrations required for effective solubilization can promote partial depolymerization or chemical modification of lignin, depending on operating conditions, which may reduce its suitability for certain material applications. Moreover, while hydrotropic systems operate at relatively low temperatures, their reliance on concentrated hydrotrope solutions introduces challenges related to chemical inventory, corrosion-resistant equipment, and water-intensive separation steps. The overall energy demand of hydrotropic extraction is therefore frequently shifted from the reactor to downstream solvent reconcentration and wastewater management, rather than eliminated (Yin et al., 2021).

DES-based extraction presents a contrasting balance of advantages and limitations. Although delignification rates may be slower and mass transfer can be hindered by the high viscosity of many DES formulations, these systems often yield lignin with higher purity and better preservation of reactive functional groups. Energy consumption in DES processes is typically distributed across moderate heating, extended residence times, and mixing requirements rather than rapid thermal input. Importantly, solvent recovery and reuse are critical determinants of DES sustainability; inefficient recycling or solvent loss can significantly increase energy and environmental costs, offsetting the benefits of mild reaction conditions. Recent assessments highlight that viscosity, solvent stability, and recyclability remain key barriers to large-scale DES implementation (Li et al., 2023).

From an application-oriented perspective, DES-extracted lignin is generally more suitable for material-focused valorization routes such as adhesives, composites, and functional fillers, where molecular uniformity and functional group availability are critical. Hydrotropic lignin, on the other hand, is particularly attractive for integrated biorefineries targeting rapid biomass fractionation and subsequent carbohydrate conversion, as well as for catalytic depolymerization routes where high delignification efficiency and aqueous compatibility are advantageous (Abolore et al., 2025). These distinctions underscore that neither extraction method can be universally defined as superior; rather, their suitability depends on aligning extraction chemistry with downstream processing and valorization objectives. **Table 3** provides a detailed comparative framework for deep eutectic solvent and hydrotropic lignin extraction, integrating mechanistic features, delignification performance, lignin structural quality, solvent recovery pathways, energy distribution, and downstream valorization compatibility.

**Table 3:** Process-level comparison of deep eutectic solvent and hydrotropic lignin extraction.

Parameter	DES extraction	Hydrotropic extraction	Representative evidence	References
Solubilization principle	Hydrogen-bonding/polarity-driven solubilization in a tunable eutectic mixture; formulation-dependent viscosity and recyclability behavior	Hydrotrope-driven lignin solubilization in concentrated aqueous acid hydrotrope; dissolution governed by hydrotrope concentration (above MHC)	DES performance and recycle difficulty/strategy discussed in SDES study; hydrotropy driven by p-TsOH concentration and dilution trigger below MHC	Li et al. (2023); Chen et al. (2017)
Typical thermal severity and time	Often moderate temperature with longer residence times; depends on viscosity and mass transfer	Mild temperature with very short residence times under optimized p-TsOH hydrotropy	Near-complete wood lignin dissolution reported at ≤80 °C for ~20 min using recyclable p-TsOH hydrotrope	Chen et al. (2017)
Delignification rate (kinetics)	Moderate, can be limited by high viscosity and diffusion; improved by formulation and mixing strategy	Very rapid delignification/dissolution under optimized p-TsOH hydrotropy	The rapid dissolution at ≤80 °C and short timescale is documented	Chen et al. (2017)
Lignin structural “fitness-for-use” trend	Often favorable for materials-oriented lignin when conditions minimize condensation; formulation can support preservation of functional groups, but outcomes are formulation-specific	Can yield relatively uncondensed lignin at low temperature, but outcomes depend on acid concentration and process window; can also alter lignin chemistry if conditions are harsh	Acid hydrotropic fractionation at low temperature can reduce condensation; material properties depend on extracted lignin state and downstream handling	Ma et al. (2020)
Lignin recovery method	Separation/recovery depends on DES formulation and recycle scheme; solvent recovery is often a major determinant of sustainability	Lignin can be precipitated by diluting spent liquor below the minimal hydrotrope concentration (MHC), enabling separation	Dissolved lignin is precipitated by diluting p-TsOH below MHC (11.5 wt%)	Wang et al. (2020)
Dominant energy drivers (system-level)	Energy can shift toward mixing (viscosity), longer residence time, and solvent recovery steps; total demand depends on recycle efficiency	Reactor heating can be modest (low temperature), but separations can dominate due to dilution/reconcentration and water evaporation load	Water evaporation/reconcentration needs are explicitly discussed as extensive in process descriptions of hydrotropy-based schemes	Zhu et al. (2023)
Chemical inventory and equipment constraints	Depends on formulation; some DES can be corrosive if acidic, but neutral/bio-based options exist; viscosity is a handling constraint	Often requires highly concentrated acid hydrotrope (e.g., p-TsOH), raising corrosion/material constraints and chemical inventory concerns	p-TsOH hydrotropy is acid-based; concentrated operation motivates corrosion-resistant systems	Chen et al. (2017)
Feedstock flexibility and robustness	High design flexibility because DES components can be tuned for different biomasses; however performance and recycling are formulation-specific	Robust delignification demonstrated across several lignocellulosic substrates, but performance depends strongly on hydrotrope concentration and process control	Rice straw hydrotropic lignin extraction and characterization provides a clear agro-residue case study	Yin et al. (2021)
Downstream valorization alignment	Typically stronger alignment with material applications when lignin functional groups and distribution are preserved and consistent; also usable for chemical routes depending on strategy	Strong for rapid fractionation integrated biorefinery designs and can support catalytic valorization; carbohydrate-rich solids can be well-suited for enzymatic hydrolysis	Process-selection reviews emphasize matching extraction to end-use and integrating separation burdens into sustainability claims	Abolore et al. (2025)

DES, deep eutectic solvent; SDES, switchable deep eutectic solvent; p-TsOH, *p*-toluenesulfonic acid; MHC, minimal hydrotrope concentration.

Collectively, these advances demonstrate that lignin extraction is no longer a purely separations-driven operation but a quality-engineering step that determines the feasibility of downstream modification and application. Once isolated, lignin must be structurally tailored to overcome inherent limitations in solubility, compatibility, and reactivity, making targeted modification strategies the next critical stage in lignin valorization.

### 4.2 Advanced lignin modification strategies for sustainable functional materials

Following extraction and fractionation, lignin must be structurally tailored to overcome inherent limitations in solubility, compatibility, and reactivity that restrict its direct use in high-value materials. Owing to its aromatic, heterogeneous, and multifunctional nature, native lignin rarely meets application requirements without further modification. Controlled lignin modification therefore represents a critical step in valorization, enabling the introduction of new reactive sites, improved processability, and enhanced interaction with polymeric matrices (Chandna et al., 2023). Contemporary lignin valorization strategies predominantly rely on two complementary molecular approaches: (i) targeted functionalization of lignin derivatives to tune reactivity and interfacial behavior, and (ii) cross-linking or network formation to generate mechanically robust and thermally stable materials. Together, these strategies convert lignin from an extracted biopolymer into an application-ready macromolecular building block.

#### 4.2.1 Targeted functionalization of lignin for improved reactivity and integration

Targeted functionalization involves the deliberate modification of lignin’s chemical structure through the introduction or transformation of functional groups such as hydroxyl, amino, or carboxyl moieties. These modifications enhance lignin’s solubility, reactivity, and compatibility with other components in polymeric and composite systems, thereby enabling more predictable structure–property relationships (Yu et al., 2023). By controlling the type and distribution of functional groups, functionalization provides the molecular foundation for subsequent cross-linking reactions and material assembly.

Enzymatic functionalization has attracted increasing interest due to its high selectivity and environmental compatibility. Enzymes such as laccases and peroxidases selectively oxidize phenolic hydroxyl groups in lignin, generating reactive radicals or quinone structures while largely preserving the native aromatic framework. Chan et al. (2019) demonstrated that laccase-mediated oxidation significantly increases the availability of reactive phenolic sites, improving lignin’s compatibility with polymer matrices. Such enzymatic routes operate under mild aqueous conditions, reducing the need for harsh reagents and aligning well with green chemistry principles.

**Figure 5:**
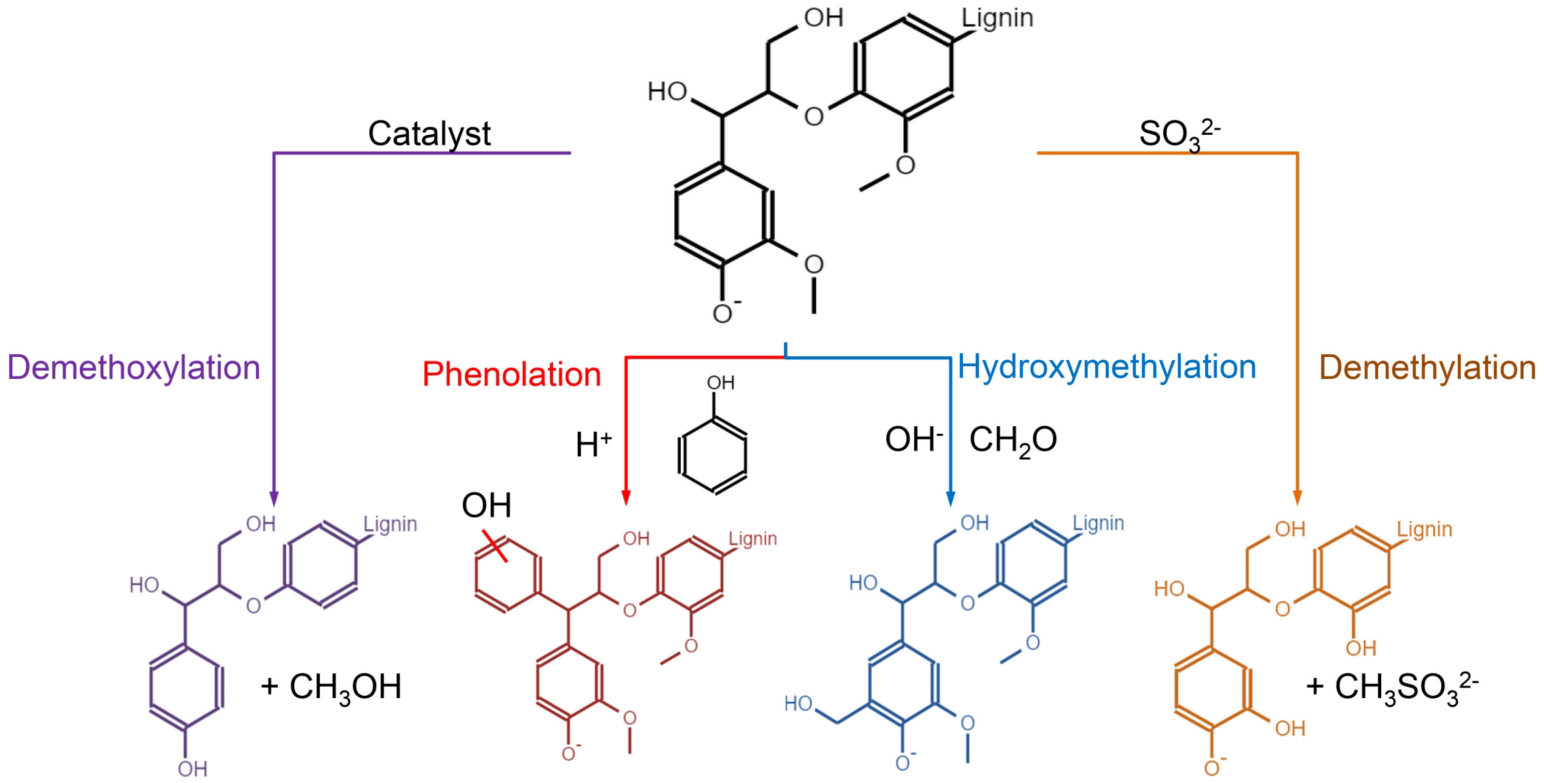
Representative chemical modification pathways for tuning lignin reactivity and functionality.

Chemical functionalization methods, by contrast, offer greater versatility and remain widely used for industrial lignin modification. Reactions such as phenolation, hydroxymethylation, demethylation, and demethoxylation are commonly employed to increase lignin reactivity and tailor its performance in polymeric systems. For example, Luo et al. (2020) reported that phenolation under acidic conditions introduces additional phenolic hydroxyl groups, markedly enhancing lignin’s suitability for phenol–formaldehyde resin synthesis. In parallel, selective fractionation during extraction—using organic solvents or DES—can produce lignin populations with controlled molecular weight, condensation degree, and functional group distribution, leading to improved solubility and thermal stability (Song et al., 2020). Representative chemical modification pathways and their effects on lignin structure are summarized in **Figure 5**.

Overall, the functionalization of lignin derivatives provides a powerful molecular toolbox for bridging plant-derived biopolymer chemistry with industrial materials engineering. By combining enzymatic selectivity with chemical versatility, researchers can generate lignin-based materials with tunable reactivity and improved processing characteristics, supporting their integration into advanced polymer and composite systems.

#### 4.2.2 Cross-linking and network formation for high-performance lignin-based polymers

Cross-linking and network formation constitute a second major modification strategy, transforming functionalized lignin into three-dimensional polymeric networks with enhanced mechanical strength, thermal stability, and chemical resistance (Grade et al., 2024). These processes involve the formation of covalent bonds either between lignin macromolecules or between lignin and complementary reactive species, enabling the production of thermosetting resins, elastomers, and composite matrices derived from renewable resources.

Conventional chemical cross-linking often relies on aldehyde-based chemistry, where hydroxymethyl intermediates react with phenolic groups on lignin to form dense network structures. Peng et al. (2023) demonstrated that such reactions effectively enhance rigidity and durability in lignin-containing phenol–formaldehyde resins. Alternative cross-linking strategies, including siloxane-based systems, have also been explored. Grade et al. (2024) reported that ester formation between lignin and siloxane molecules significantly improves tensile strength and elasticity in lignin–silicone composites, illustrating lignin’s adaptability to diverse polymer architectures.

Enzymatic cross-linking offers a greener alternative by exploiting oxidative coupling reactions catalyzed by enzymes such as laccase. These enzymes promote intermolecular bonding between phenolic units under mild conditions, eliminating the need for toxic cross-linking agents and improving the environmental profile of the resulting materials (Hassani et al., 2013). In addition, emerging studies have shown that DES can facilitate cross-linking by enhancing lignin solubility and reactivity through hydrogen-bonding interactions. Such DES-assisted systems have been reported to produce more uniform polymer networks and enable cross-linking under relatively mild processing conditions, further expanding the toolbox for sustainable lignin-based material design (Lin et al., 2018).

In summary, cross-linking and network formation strategies are essential for converting functionalized lignin into high-performance materials suitable for structural and functional applications. When integrated with targeted functionalization approaches, these strategies enable the rational design of lignin-based polymers, composites, and coatings that combine renewable origin with competitive performance. These modified lignin materials form the basis for the application-focused developments discussed in the following section.

## 5 Lignin-based materials for sustainable functional systems

Lignin has evolved from being an industrial byproduct to a strategic biopolymer at the center of sustainable materials science. Its aromatic structure, abundance, and chemical versatility make it a renewable feedstock for producing functional materials spanning energy storage, construction, polymer science, additive manufacturing, and nanotechnology. Building on advances in lignin fractionation and targeted modification, lignin can now be designed as (i) a carbon precursor, (ii) a nanoscale functional filler, (iii) a reactive macromolecule for adhesive/polymer networks, and (iv) a performance additive in commodity systems. The following subsections summarize key lignin-derived material classes and emphasize where performance is already competitive and where limitations remain linked to lignin heterogeneity and interfacial compatibility.

### 5.1 Lignin-derived carbon materials are benchmarked against PAN-based systems

Lignin has been intensively studied as a renewable precursor for carbon fiber production because it is abundant, aromatic, and available in large quantities as a byproduct from kraft pulping and emerging biorefinery operations. However, the scientific and industrial relevance of lignin-derived carbon fibers must be evaluated through quantitative benchmarking against polyacrylonitrile (PAN)-based carbon fibers, which dominate commercial structural carbon fiber markets and set the performance standard. PAN-based carbon fibers are widely reported to reach tensile strengths on the order of several gigapascals, with conventional PAN-derived fibers achieving tensile strengths as high as approximately 7 GPa in high-performance grades (Alarifi et al., 2016; Soulis et al., 2020). This high tensile strength is closely linked to the ability of PAN precursor fibers to develop strong molecular orientation during spinning and to form ordered carbon structures after stabilization and carbonization.

When lignin is used as the primary precursor, the reported tensile strengths of resulting carbon fibers are typically substantially lower than PAN-based fibers, and the literature consistently shows that lignin-derived fibers occupy a different application space unless special strategies are used. The pioneering work by Kadla and co-workers demonstrated the feasibility of producing carbon fibers from commercially available kraft lignin and documented the key processing challenges that limit mechanical performance, including spinnability, stabilization behavior, and defect formation (Kadla et al., 2002). Subsequent reviews and process-focused studies reinforce that lignin’s intrinsic heterogeneity, broad molecular-weight distribution, and irregular branching constrain chain alignment and promote microstructural defects during thermal conversion, which directly reduces tensile strength compared with PAN-derived fibers (Baker and Rials, 2013; Fang et al., 2017). For this reason, lignin-derived carbon fibers are often reported in the sub-GPa to low-GPa tensile strength range depending on lignin type, fractionation, spinning route (melt spinning, electrospinning, solution spinning), stabilization protocol, and carbonization conditions, with improvements commonly achieved through fractionation or chemical modification that narrows lignin dispersity and improves spinnability and stabilization behavior (Jia et al., 2023).

Quantitative benchmarking therefore supports a clear interpretation: lignin-derived carbon fibers are not yet a drop-in substitute for high-performance PAN fibers in aerospace-grade structural roles, but they can be technically and economically compelling for applications where the dominant constraints are cost, sustainability, conductivity, or moderate mechanical reinforcement rather than maximum tensile strength. In this context, a major driver is the cost structure of PAN fibers, where the PAN precursor is frequently identified as a significant contributor to the total fiber cost; by contrast lignin feedstock is low value or treated as a byproduct in many industrial settings (Baker and Rials, 2013). Mechanistically, closing the performance gap requires addressing lignin precursor consistency and molecular organization, because tensile strength in carbon fibers is highly sensitive to defect density, microstructural alignment, and stabilization-controlled morphology, as similarly demonstrated for other bio-derived carbon nanofibers where prolonged thermal treatment drives irreversible structural transformations (Nuge et al., 2024). Practical strategies emphasized in the literature include lignin fractionation to reduce polydispersity, chemical modification to tune softening behavior and suppress defects, blending with spinnable polymers to enhance precursor fiber integrity, and stabilization protocols that minimize fusion and pore formation (Fang et al., 2017; Jia et al., 2023). This evidence-based benchmarking reframes lignin-derived carbon fibers as an application-targeted, sustainability-driven platform that is most credible in large-volume markets where moderate performance is sufficient and where the economic and environmental burdens of PAN-based fibers are limiting.

### 5.2 Lignin-based nanomaterials enable multifunctional composites and coatings

Building on its potential as a carbon precursor, lignin’s transformation into nanoscale architectures has significantly expanded its application space, particularly in multifunctional composites and surface coatings. Lignin-based nanomaterials—most notably lignin nanoparticles (LNPs) and nanofibrils—have emerged as versatile, bio-based building blocks whose properties derive from lignin’s aromatic backbone, chemical functionality, and renewable origin. Nanoscale structuring increases lignin’s specific surface area, dispersibility, and accessibility of functional groups, thereby enhancing its chemical reactivity and compatibility with polymer matrices, thin films, and coating systems (Ruwoldt et al., 2023).

A range of fabrication strategies has been developed to produce lignin nanomaterials, including solvent exchange, ultrasonication, mechanical shearing, and rapid freezing, each influencing particle size, morphology, and surface chemistry. Zhang et al. (2021) demonstrated that these approaches yield diverse nanostructures—such as colloidal spheres and nanofibers—with controlled size distributions, leading to measurable improvements in thermal stability and mechanical reinforcement when incorporated into polymer composites. In particular, nanoparticles stabilized through π–π interactions showed enhanced solubility and uniform dispersion, which is critical for achieving homogeneous composites and strong interfacial bonding. Similarly, Lizundia et al. (2021) reported multifunctional lignin-based nanocomposites that combined mechanical reinforcement with intrinsic UV resistance, illustrating the suitability of LNPs as functional fillers in packaging, electronics, and environmentally responsive materials.

Beyond bulk composites, lignin-derived nanomaterials have gained increasing attention in coating technologies, where both structural reinforcement and active functionality are required. Li et al. (2018) employed vinyl-silylated lignin to fabricate coatings with improved toughness and thermal resistance, while Shikinaka et al. (2020) incorporated nanoparticulated lignin into polymer films to enhance UV absorption and tensile performance. These studies demonstrate that lignin nanomaterials can simultaneously act as reinforcing agents and active protective components, enabling coatings with improved durability and multifunctionality.

Despite these promising advances, several challenges continue to limit large-scale deployment. Variability in lignin structure arising from botanical source and extraction method translates directly into differences in nanoparticle composition, surface chemistry, and performance (Fazeli et al., 2024). In addition, scaling nanoparticle production while maintaining uniformity, colloidal stability, and reproducibility remains a key technical and economic barrier, highlighting the need for optimized synthesis protocols and continuous or flow-based processing strategies. Addressing these issues is essential for translating laboratory-scale demonstrations into industrially viable technologies.

Overall, the integration of lignin nanoparticles and nanofibrils into nanocomposites and coatings represents a significant advance in sustainable materials science. These bio-derived nanostructures not only reduce dependence on petroleum-based additives but also impart enhanced mechanical, thermal, optical, and protective functionalities to end-use products (Mandal et al., 2023). As nanofabrication and functionalization techniques continue to mature, lignin-based nanomaterials are well positioned to play a central role in the development of eco-efficient, high-performance materials aligned with circular bioeconomy objectives.

### 5.3 Lignin-based adhesives and binders for sustainable wood and composite manufacturing

Lignin-based adhesives and binders have attracted growing attention as eco-friendly alternatives to synthetic resins traditionally used in wood and composite manufacturing. Derived from a natural and renewable source, lignin offers an opportunity to reduce the carbon footprint and toxic emissions associated with petroleum-based adhesives such as phenol–formaldehyde and urea–formaldehyde resins. As a major byproduct of the pulp and paper industry, lignin is readily available, biodegradable, and rich in phenolic structures that provide inherent adhesive potential and chemical reactivity (Borges et al., 2022). The development of lignin-derived adhesives aligns with the broader goal of promoting sustainable materials that integrate high performance with environmental compatibility. The adhesive performance of lignin largely depends on its molecular structure, which varies with the extraction and purification method. Yang et al. (2023) examined the use of lignins with different degrees of condensation as wood adhesives, revealing that slightly condensed or chemically protected lignins achieved bonding strengths above 0.7 MPa, meeting industrial standards for structural wood products. These findings highlight the importance of controlling lignin’s chemical reactivity and condensation level to optimize its adhesive efficiency. Similarly, Podschun et al. (2016) demonstrated that phenolated lignins—produced by introducing phenolic hydroxyl groups—exhibited improved bonding performance in veneer and particleboard applications. Phenolation enhances the cross-linking ability of lignin, making it more reactive toward formaldehyde or other aldehydes, thus enabling stronger and more durable bonds while maintaining lower environmental impact. In composite manufacturing, lignin has been effectively integrated into phenol-formaldehyde and bio-based resin systems. Zhao et al. (2016) prepared lignin–phenol–formaldehyde adhesives that demonstrated superior bonding strength and durability compared to conventional phenol–formaldehyde resins. The inclusion of lignin not only reduces phenol consumption—a petroleum-derived compound—but also enhances the adhesive’s heat resistance and long-term stability. Moreover, Norström et al. (2018) explored the development of green binders combining lignin and tannins, achieving comparable bonding performance to traditional adhesives while reducing formaldehyde emissions and overall environmental impact (Aristri et al., 2021). These hybrid systems demonstrate the synergistic potential of natural polyphenols in producing adhesives that balance sustainability and functionality. Despite these advances, several challenges hinder the large-scale commercialization of lignin-based adhesives. The variability in lignin composition, influenced by biomass source and isolation process, leads to inconsistencies in adhesive performance. Additionally, the extraction and purification of lignin can be energy-intensive and costly, limiting its economic competitiveness with established synthetic adhesives (Yang et al., 2023). Further, achieving consistent bonding strength and moisture resistance remains a technical hurdle, particularly for outdoor or load-bearing applications where durability under varying environmental conditions is critical (Aristri et al., 2021; Schagerl et al., 2022). To address these limitations, research is increasingly focusing on chemical modification, enzymatic functionalization, and hybrid formulations that combine lignin with other bio-based polymers to improve adhesion and processing efficiency. Overall, lignin-based adhesives and binders represent a promising pathway toward sustainable, low-emission adhesive technologies for wood and composite industries. Their potential to replace or partially substitute petroleum-based resins contributes to reducing environmental impact while supporting the development of circular bioeconomy systems. Continued innovation in lignin modification chemistry, process optimization, and performance enhancement will be essential to fully realize lignin’s role as a renewable and cost-effective component in next-generation adhesive formulations (Norström et al., 2018). Complementary to nanomaterial development, lignin’s intrinsic phenolic reactivity has positioned it as a viable component in bio-adhesive systems designed to replace formaldehyde-based resins.

### 5.4 Lignin-based bioplastics: quantitative comparison with fossil-derived polymers

Lignin-based bioplastics have been proposed as renewable alternatives or modifiers for petroleum-derived polymers, but their practical feasibility depends on quantitative comparison with the mechanical property envelope of conventional plastics and on how lignin affects strength–ductility trade-offs, a challenge that is also widely reported for other bio-based polymer systems such as thermoplastic starch composites (Fazeli, 2018). Commodity polyolefins such as polyethylene and polypropylene are widely used because they combine moderate tensile strength with high elongation at break and robust processability. Open-access experimental studies on polyethylene clearly show tensile strengths in the tens of megapascals with strong dependence on grade and processing, alongside substantial ductility that supports high elongation at break in many applications (Amjadi and Fatemi, 2020). For low-density polyethylene films used in practical applications, reported tensile strength values are commonly at least ~10 MPa with elongation at break often above ~200%, illustrating the high-ductility baseline that lignin-containing blends must be compared against when intended for flexible packaging-type functions (Szlachetka et al., 2021).

Against this benchmark, lignin incorporation often yields materials with tensile strengths that can remain in a comparable order of magnitude but with a pronounced tendency toward reduced elongation at break unless compatibilization or plasticization strategies are used. A well-cited quantitative case is PLA–lignin bioplastics, where lignin is introduced into a relatively stiff biopolymer matrix. Spiridon and co-workers evaluated PLA–lignin bioplastics and showed that lignin addition modifies mechanical and durability-related properties, while accelerated weathering data emphasize that performance changes are application-dependent and closely linked to formulation and interfacial structure (Spiridon et al., 2015). More broadly, a major high-authority review on chemical modification of lignins explains why lignin commonly increases stiffness and brittleness and why compatibilization or lignin functionalization is often necessary to improve interfacial adhesion and restore ductility in polymer blends and composites (Laurichesse and Avérous, 2014). These sources align with the consistent trend reported across lignin–polymer systems: lignin can contribute to strength retention and thermal/UV/antioxidant functionality, but ductility and toughness frequently decrease because of polarity mismatch, aggregation, and weak interfaces unless lignin is modified or coupling agents are used (Laurichesse and Avérous, 2014).

Lignin-containing bioplastics and biocomposites can achieve strength levels relevant to many rigid or semi-rigid applications, but they typically struggle to match the extreme elongation and toughness envelope of commodity polyolefins without formulation engineering. The most realistic near-term positioning is not wholesale replacement of PE or PP, but targeted substitution or functional upgrading where lignin’s aromatic structure provides measurable benefits such as UV shielding, oxidative stability, antioxidant activity, and increased stiffness, and where reduced ductility is acceptable or can be mitigated through compatibilization chemistry. This interpretation is consistent with the broader lignin polymer literature, which emphasizes that performance is controlled less by lignin presence per se than by lignin type, molecular weight distribution, purification/fractionation history, chemical modification, and interfacial design (Laurichesse and Avérous, 2014; Spiridon et al., 2015).

### 5.5 Lignin as sustainable additive and functional component in cement and concrete systems

The incorporation of lignin into cementitious materials has emerged as a promising strategy for reducing the environmental footprint of the construction sector while maintaining or enhancing concrete performance. As one of the most carbon-intensive industries globally, cement production presents a critical target for sustainability-driven innovation. Lignin, as a renewable and widely available biopolymer, can fulfill multiple functional roles in cement and concrete systems—including partial cement replacement, plasticization, grinding aid activity, and hydration control—thereby contributing to both carbon mitigation and performance optimization (Dey et al., 2025). The use of lignin-based additives aligns directly with circular bioeconomy principles by valorizing industrial byproducts and reducing dependence on energy- and emission-intensive clinker production.

Partial replacement of Portland cement with lignin-rich ash or modified lignin derivatives has been shown to lower the overall environmental impact of concrete formulations. Lignin-containing materials can interact with calcium hydroxide and other hydration products, promoting pozzolanic reactions that contribute to strength development and matrix densification (Nair et al., 2024). By reducing clinker content—the dominant source of CO_2_ emissions in cement manufacturing—this substitution strategy directly decreases the carbon intensity of concrete while preserving mechanical performance. In addition, lignin-based materials can function as cement extenders, improving resource efficiency without compromising structural integrity.

Among lignin’s most established roles in cement technology is its function as a plasticizer or water-reducing agent. Lignin-derived plasticizers, particularly those originating from pulp and paper mill byproducts, enhance cement particle dispersion and improve the flowability of fresh concrete (Ali et al., 2026). By lowering the required water-to-cement ratio, these additives promote denser microstructures, higher compressive strength, and improved durability. Lignosulfonates are especially effective due to their anionic character and surface activity, which induce electrostatic repulsion between cement particles, preventing agglomeration and segregation during mixing.

Beyond rheology control, lignin can also act as a grinding aid during cement production. Small additions of lignin or sulfonated lignin derivatives reduce particle agglomeration during milling, enabling finer grinding with lower energy consumption (Carvalho et al., 2023). This contributes to improved production efficiency and reduced electricity demand, further enhancing the sustainability profile of cement manufacturing. Moreover, lignin-based additives have been reported to influence hydration kinetics and hardened paste microstructure, resulting in compressive strength increases of 30–60% under distilled water curing and 2–20% under seawater exposure (Akond and Lynam, 2020). These findings highlight lignin’s ability to improve both mechanical performance and environmental resilience, particularly in aggressive service conditions.

Chemical modification further broadens lignin’s applicability in cement systems. Sulfonation introduces sulfate groups into the lignin structure, increasing zeta potential and enhancing dispersant efficiency, as demonstrated by Carvalho et al. (2023). The resulting improvement in particle dispersion leads to reduced viscosity and more uniform setting behavior. In addition, lignin-based additives have been explored as set retarders, effectively slowing hydration reactions and extending setting times (Kampragkou et al., 2024). This functionality is particularly valuable in mass concreting applications, where heat evolution control is critical, and in environments exposed to moisture fluctuations or acidic conditions.

Overall, lignin’s multifunctional behavior—as a cement substitute, dispersant, grinding aid, and hydration modifier—positions it as a versatile and technically credible component in next-generation cementitious materials. Its incorporation addresses both environmental and engineering challenges by reducing carbon emissions while enhancing workability, strength development, and durability. Continued research focused on lignin molecular design, dosage optimization, and compatibility with cement hydration chemistry will be essential to fully realize its industrial potential. Within a broader bio-based materials framework, lignin-based additives exemplify how renewable macromolecules can be integrated into traditionally mineral-dominated systems to advance sustainable construction practices.

### 5.6 Lignin-based composites for sustainable 3D printing applications

The integration of lignin into additive manufacturing materials represents an emerging strategy for advancing sustainable, bio-based polymers and composite filaments. As demand grows for environmentally responsible materials with tailored mechanical and thermal performance, lignin has attracted increasing attention due to its renewability, aromatic structure, and intrinsic chemical functionality. Successful 3D printing materials must satisfy stringent requirements including stable melt flow or resin viscosity, dimensional accuracy, and mechanical integrity after deposition. Owing to its oxygenated aromatic backbone, β–O–4′ linkages, and aliphatic ether functionalities, lignin offers molecular features that can contribute to these requirements while reducing reliance on petroleum-derived polymers (Nguyen et al., 2018a). When judiciously blended or chemically modified, lignin can act as both a structural component and a functional modifier in printable polymer systems.

Recent studies have demonstrated multiple formulation strategies for lignin-based composites compatible with additive manufacturing. Huang et al. (2019) reported a coordination-bonding approach in which Zn-based interactions between lignin nanoparticles and elastomer matrices created sacrificial energy-dissipating linkages. This strategy improved lignin dispersion and significantly enhanced toughness and ductility, enabling printable composites with lignin loadings up to 30 wt%. These results highlight the importance of interfacial engineering in overcoming lignin aggregation and brittleness, two key challenges in polymer–lignin systems.

A wide range of thermoplastic–lignin composites have been developed for extrusion-based printing techniques such as fused filament fabrication. Gkartzou et al. (2017) demonstrated that PLA/organosolv lignin blends containing up to 40 wt% lignin exhibited stable extrusion behavior, acceptable thermal properties, and reduced material cost. Similarly, Obielodan et al. (2022) incorporated 40 wt% organosolv lignin into ABS matrices reinforced with short carbon fibers, producing printed components with enhanced mechanical strength. For photopolymer-based printing, Zhang et al. (2019) reported that adding 0.2–1.0 wt% softwood kraft lignin to methacrylate resins used in stereolithography increased tensile strength by up to 58%, with optimal performance at approximately 0.4 wt% lignin. Collectively, these studies demonstrate that lignin can function as both a reinforcing filler and a performance modifier across multiple additive manufacturing platforms. Additional quantitative examples—including PLA-based wood–plastic composites and chemically modified lignin–PLA systems—are summarized in **Table 4**, underscoring lignin’s role in enhancing printability, mechanical integrity, and sustainability.

**Table 4:** Representative lignin-based composite systems and their performance in 3D printing applications.

Lignin type	Polymer/resin matrix	Key findings	References
Organosolv lignin	PLA	Up to 40 wt% lignin improved mechanical and thermal properties of FFF-printed specimens	Gkartzou et al. (2017)
Organosolv lignin	ABS with carbon fibers	Lignin loadings up to 40 wt% enhanced mechanical performance relative to neat ABS composites	Nguyen et al. (2018b)
Alkali lignin	PLA	Low lignin addition (≤0.5 wt%) increased PLA toughness (~82%) and imparted UV-blocking and antioxidant functionality	Obielodan et al. (2022)
COOH-functionalized lignin	PLA	Carboxylation improved interfacial adhesion and enabled cost reduction in PLA filaments	Hong et al. (2021)
Softwood kraft lignin	SLA	Lignin addition increased tensile strength by 46–64% in photopolymerized composites	Zhang et al. (2019)
Lignin-based ink (native lignocellulosic component)	Triblock copolymer–crosslinked ink	Printed lignin structures showed high wet tensile strength (~30 MPa) and improved thermal and water stability	Jiang et al. (2020)
Organosolv lignin (OPEFB-derived)	PLA	Low lignin content (3–5 wt%) improved modulus and interlayer adhesion; ≥10 wt% reduced tensile strength	Zaidi et al. (2023)
Spruce-derived lignin	PLA	Lignin acted as a nucleating agent for PLA crystallization; high lignin loadings enhanced antioxidant activity	Tanase-Opedal et al. (2019)
Organosolv lignin (wood-industry waste)	Acrylic resin/PANI composite	Lignin improved hardness, wettability, and electrical conductivity in printed composites	Arias-Ferreiro et al. (2022)
LMA	DLP	Lignin-enabled transesterification yielded self-healing, recyclable, bio-based printable resins	Johnson et al. (2024)

PLA, polylactic acid; ABS, acrylonitrile–butadiene–styrene; SLA, stereolithography; DLP, digital light processing; PANI, polyaniline; OPEFB, oil palm empty fruit bunch; FFF, fused filament fabrication; COOH, carboxyl functional group; LMA, methacrylated lignin.

Beyond PLA-based formulations, lignin has been successfully integrated into other thermoplastic systems. In nylon–lignin composites, organosolv hardwood lignin acted as a reinforcing phase that reduced melt viscosity while increasing stiffness, thereby improving printability (Wu et al., 2025b). These effects were attributed to hydrogen bonding between lignin domains and the polymer matrix, which enhanced interfacial adhesion. In PLA systems containing 20–40 wt% lignin, crystallization behavior was significantly altered, with lignin acting as a nucleating agent that improved extrudability and flow uniformity, as confirmed by thermogravimetric analysis, X-ray diffraction, and scanning electron microscopy (Tanase-Opedal et al., 2019). Conversely, acetylation of alkali and organosolv lignins reduced hydroxyl-group density and self-aggregation, leading to improved compatibility with PLA, higher elongation at break, and enhanced thermal stability (Gordobil et al., 2014).

**Figure 6:**
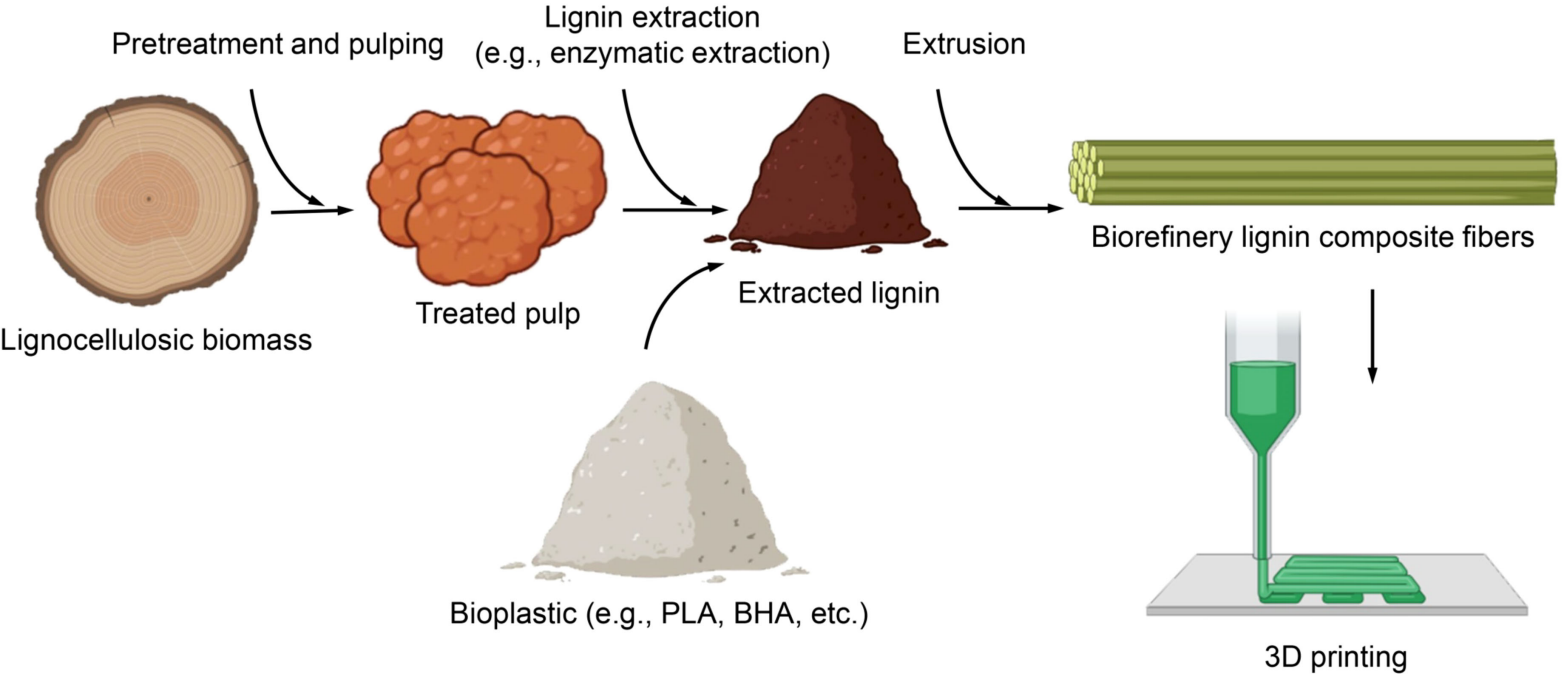
Schematic illustration of the integration of biorefinery-derived lignin into polymer composites for additive manufacturing.

Lignin has also been incorporated into photoactive resins and biopolyester matrices for advanced additive manufacturing applications. Acylated organosolv lignin was successfully blended into acrylate-based photoresins at loadings up to 15 wt%, yielding materials with high ductility, excellent print resolution, and uniform layer fusion, albeit with slightly reduced thermal stability compared to conventional resins (Sutton et al., 2018). Vaidya et al. (2019) reported that blending 20 wt% biorefinery lignin derived from *Pinus radiata* with polyhydroxybutyrate (PHB) improved surface finish and reduced warping by 38–78% relative to neat PHB. The enhanced dimensional stability was attributed to lignin’s irregular molecular architecture, which lowers interfacial tension and suppresses shrinkage during cooling and solidification. The integrated workflow from lignin extraction to composite fabrication and 3D printing is schematically illustrated in **Figure 6**.

Overall, lignin-based composites for additive manufacturing demonstrate that lignin can transition from an industrial byproduct to a high-value functional component in advanced fabrication technologies. Through targeted chemical modification, nanoparticle engineering, and polymer blending, lignin can improve stiffness, toughness, thermal behavior, and sustainability metrics in printable materials. While challenges related to batch-to-batch variability, rheological control, and long-term durability remain, the growing body of quantitative evidence supports lignin’s realistic positioning as a cost-effective and sustainable additive for next-generation 3D printing materials within a circular manufacturing paradigm.

### 5.7 Emerging applications of lignin in next-generation sustainable technologies

Lignin is increasingly recognized as a pivotal biopolymer in the development of next-generation sustainable technologies, with applications extending beyond established material uses toward multifunctional and high-value systems. As a complex aromatic macromolecule and one of the most abundant renewable carbon resources, lignin represents an underexploited feedstock for advanced functional materials, hybrid systems, and niche chemical applications that cannot be readily addressed by conventional biopolymers (Vasile and Baican, 2023). Recent progress in green chemistry, nanotechnology, and molecular engineering has enabled lignin-derived platforms to enter emerging domains such as energy storage, biomedical materials, environmental remediation, and smart functional systems, reflecting a shift from bulk substitution toward value-driven utilization (Khan et al., 2024).

In contrast to the mature applications discussed above, emerging lignin technologies are characterized by their reliance on precise structural control, targeted functionalization, and application-specific performance rather than volume-driven deployment. These systems often exploit lignin’s intrinsic aromaticity, redox activity, UV absorption, antioxidant behavior, and surface functionality rather than its bulk mechanical contribution. Examples include lignin-derived electrode materials and redox-active components for energy storage, antioxidant and UV-shielding additives for biomedical or packaging applications, and functional fillers for adsorption, catalysis, or sensing platforms. In these contexts, lignin’s heterogeneous structure becomes a tunable asset when appropriately engineered, rather than a limitation. The technological foundation of many emerging applications lies in controlled modification and partial depolymerization strategies that expose or generate functional motifs without fully converting lignin into low-molecular-weight monomers (Zhang et al., 2023). Chemical modification routes such as amination, sulfonation, hydroxyalkylation, nitration, urethanization, phenolation, alkylation, and etherification are increasingly employed to tailor solubility, interfacial compatibility, and reactivity for specialized functions (Chen et al., 2025). These approaches differ fundamentally from bulk depolymerization routes discussed in Section 5.8, as they aim to preserve lignin’s macromolecular character while selectively enhancing specific properties relevant to advanced functional systems.

At the same time, emerging applications face substantial barriers related to reproducibility, scalability, and cost-performance balance. Many high-functionality lignin-based systems remain at laboratory or pilot scale, where performance advantages can be demonstrated but economic competitiveness with fossil-based or established bio-based alternatives is not yet assured (Ismail et al., 2022). As a result, realistic application niches are typically defined by functional differentiation, regulatory drivers, or sustainability premiums rather than direct cost parity. This distinction underscores the importance of critically benchmarking lignin-based technologies against incumbent materials, not only in terms of performance metrics but also with respect to maturity, limitations, and system-level trade-offs.

To contextualize the practical maturity and technological positioning of these emerging systems, **Table 5** provides a concise quantitative benchmarking of representative lignin-derived materials against fossil-based counterparts. It highlights achievable performance ranges, primary technical limitations, and realistic application niches, offering a structured framework for distinguishing near-term opportunities from longer-term research directions. This benchmarking perspective reinforces the view that lignin’s future impact will arise not from a single dominant application, but from a portfolio of targeted technologies that leverage its unique molecular features where they provide clear functional or sustainability advantages.

**Table 5:** Quantitative benchmarking of lignin-based materials versus fossil-derived benchmarks.

Application	Fossil-based benchmark	Typical performance (fossil)	Lignin-based counterpart	Typical performance (lignin-based)	Performance gap and primary limitation	References
Carbon fibers	PAN-based carbon fibers	Tensile strength ≈ 3–7 GPa; high modulus	Lignin-derived carbon fibers	Tensile strength ≈ 0.3–1.5 GPa	≈10–30% of PAN strength; limited by lignin heterogeneity, poor molecular orientation, and stabilization-induced defects	Kadla et al. (2002); Baker and Rials (2013); Fang et al. (2017)
Structural plastics	Polyethylene/polypropylene	Tensile strength ≈ 20–40 MPa; high elongation	Lignin-based bioplastics/blends	Tensile strength ≈ 20–50 MPa; low elongation	Strength comparable in rigid systems; brittleness and poor ductility limit flexible applications	Laurichesse and Avérous (2014); Spiridon et al. (2015)
Adhesives	Phenol–formaldehyde resins	High bonding strength; fossil phenol	Lignin-substituted phenolic adhesives	Comparable bonding strength at partial substitution	Reactivity depends on lignin structure; condensation reduces performance at high substitution levels	Podschun et al. (2015); Zhao et al. (2017)
Cement additives	Synthetic plasticizers	Improved flow and strength	Lignosulfonates	Comparable water reduction and dispersion	Performance sensitive to molecular weight and sulfonation degree	Carvalho et al. (2018); Aïtcin (2000)
3D printing polymers	PLA/ABS	High printability; stable melt flow	Lignin-filled filaments	Reduced melt flow; improved stiffness	Printability limited by viscosity and dispersion at high lignin loadings	Gkartzou et al. (2017); Hong et al. (2021)

PAN, polyacrylonitrile; PLA, polylactic acid; ABS, acrylonitrile–butadiene–styrene; GPa, gigapascal; MPa, megapascal.

### 5.8 Catalytic depolymerization of lignin toward aromatic chemicals and monomers

While lignin-based materials represent a major valorization pathway, the catalytic depolymerization of lignin into low-molecular-weight aromatic chemicals constitutes an equally important and complementary strategy within the lignin value chain. From a circular bioeconomy perspective, this pathway aims at the selective cleavage of lignin’s complex polymeric network to generate renewable aromatic monomers that can substitute petroleum-derived phenols, benzene–toluene–xylene (BTX) compounds, and functional intermediates for polymers, resins, and fine chemicals (Rinaldi et al., 2016; Schutyser et al., 2018). Unlike material-oriented routes that preserve lignin’s macromolecular structure, depolymerization strategies intentionally target interunit linkages—particularly β–O–4′ ether bonds—to unlock lignin’s intrinsic chemical potential at the molecular level (Zakzeski et al., 2010).

Catalytic depolymerization approaches can be broadly classified into reductive, oxidative, and solvolytic routes, each characterized by distinct reaction mechanisms and product distributions. Among these, reductive catalytic fractionation (RCF) has emerged as one of the most extensively studied platforms, combining biomass fractionation with *in situ* lignin depolymerization to produce stabilized phenolic monomers while preserving a carbohydrate-rich solid fraction (Renders et al., 2017; Abu-Omar et al., 2021). RCF processes typically employ supported metal catalysts such as Ru, Ni, or Pd in alcohol solvents under hydrogen or hydrogen-donor conditions, yielding alkylated phenols with reduced recondensation propensity (Schutyser et al., 2018). Although these monomers are attractive precursors for polymer synthesis and chemical manufacturing, solvent recovery, catalyst stability, and hydrogen demand remain key challenges for large-scale deployment (Abu-Omar et al., 2021).

Oxidative depolymerization represents an alternative strategy, targeting the controlled oxidation of lignin to generate aromatic aldehydes, acids, and ketones such as vanillin, syringaldehyde, and aromatic carboxylic acids, which are used in flavoring, pharmaceuticals, and specialty chemicals (Zakzeski et al., 2010; Behling et al., 2016). However, oxidative routes frequently suffer from over-oxidation, limited selectivity, and complex downstream separation requirements, which hinder scalability. Solvolytic and acid-catalyzed depolymerization methods, including hydrothermal and alcoholysis processes, offer simpler reaction systems but often generate highly complex product mixtures that require energy-intensive purification, reducing their overall sustainability (Schutyser et al., 2018).

From a systems perspective, catalytic depolymerization routes are most compelling when they target high-value, low-volume chemicals that justify the associated energy, solvent, and catalyst inputs. Life-cycle and techno-economic analyses increasingly emphasize that depolymerization pathways must be evaluated not only against fossil-derived aromatic production, but also against the incumbent practice of lignin combustion for energy recovery (Renders et al., 2017; Abu-Omar et al., 2021). Environmental and economic performance is highly sensitive to catalyst lifetime, solvent recycling efficiency, hydrogen sourcing, and achievable monomer yields. Consequently, catalytic depolymerization should be viewed not as a universal solution for lignin valorization, but as a targeted strategy best suited for applications where renewable aromatics offer clear functional, regulatory, or sustainability advantages.

Importantly, chemical and material valorization pathways are not mutually exclusive. Integrated biorefinery concepts increasingly envision parallel lignin utilization routes, in which a fraction of lignin is depolymerized to chemicals while the remaining lignin is upgraded into functional materials such as carbon fibers, resins, or composites (Rinaldi et al., 2016; Schutyser et al., 2018). Such hybrid strategies maximize value extraction from lignin and highlight the need for flexible, application-driven valorization frameworks rather than single-pathway optimization.

## 6 Environmental and economic implications of lignin utilization

The utilization of lignin presents substantial environmental and economic opportunities that align closely with global strategies for circularity, decarbonization, and sustainable industrial development. As a major byproduct of the pulp and paper, agricultural, and biofuel industries, lignin constitutes an abundant, renewable, yet historically underutilized resource. Transforming lignin from an industrial residue into a value-added feedstock offers a pathway to reduce dependence on fossil-derived materials while simultaneously lowering waste generation and greenhouse gas emissions (Trezza et al., 2025). Traditionally combusted for low-value energy recovery, lignin is increasingly being repositioned as a sustainable raw material for bioplastics, adhesives, carbon fibers, construction materials, fuels, and specialty chemicals. This transition supports circular economy principles by extending material lifetimes, enhancing resource efficiency, and improving the overall sustainability of integrated biorefinery systems (Vasile and Baican, 2023).

Beyond waste reduction, lignin utilization can contribute to climate change mitigation through carbon retention in durable products such as composites, carbon fibers, and construction materials. These lignin-derived products store biogenic carbon over extended service lifetimes, thereby delaying atmospheric carbon release relative to direct combustion (Yan et al., 2025). Substituting petroleum-derived polymers and aromatics with lignin-based alternatives can further reduce life-cycle greenhouse gas emissions, particularly when conversion pathways rely on low-carbon energy and efficient processing. Locally sourced lignin also reduces transportation-related emissions and strengthens regional bioeconomy supply chains. In addition, lignin’s inherent biodegradability and antioxidant functionality can reduce the persistence and environmental impact of polymer waste, contributing to broader pollution-mitigation goals (Song et al., 2026).

The economic implications of lignin valorization are equally significant. Converting lignin into higher-value products enables pulp, paper, and biofuel producers to diversify revenue streams, improve process economics, and reduce reliance on commodity energy markets. Valorization strategies can also offset costs associated with lignin disposal and recovery while creating new markets for advanced bio-based chemicals and functional materials (Abbati de Assis et al., 2018). Recent advances in thermochemical, biological, and electrochemical conversion technologies have improved process efficiencies and expanded the portfolio of lignin-derived products, strengthening the economic case for integrated biorefineries (John et al., 2025).

Lignin utilization further supports socioeconomic sustainability, particularly in agricultural and rural regions. Recovering lignin from agricultural residues and forestry byproducts creates additional income streams, promotes rural industrial development, and encourages adoption of bio-based production systems. Moreover, lignin-based additives that enhance UV resistance, thermal stability, and mechanical durability can extend product lifetimes and reduce material consumption over time (Lv et al., 2023). Collectively, these environmental, economic, and social benefits position lignin as a strategic resource for achieving sustainability goals that integrate environmental protection with economic resilience.

Nevertheless, several technical and operational challenges continue to constrain large-scale deployment. Lignin’s intrinsic heterogeneity and complex molecular structure complicate fractionation, purification, and reproducible conversion into uniform products. Wang et al. (2019) emphasized the importance of advanced analytical methods for tracking lignin transformations during chemical upgrading. Biological valorization routes also face challenges associated with inhibitory lignin-derived compounds that limit microbial activity, requiring improved biocatalyst selection and metabolic pathway engineering (Mandal et al., 2023). Despite these constraints, continued advances in catalysis, process integration, and bioengineering are steadily expanding the feasible application space for lignin-based technologies.

**Figure 7:**
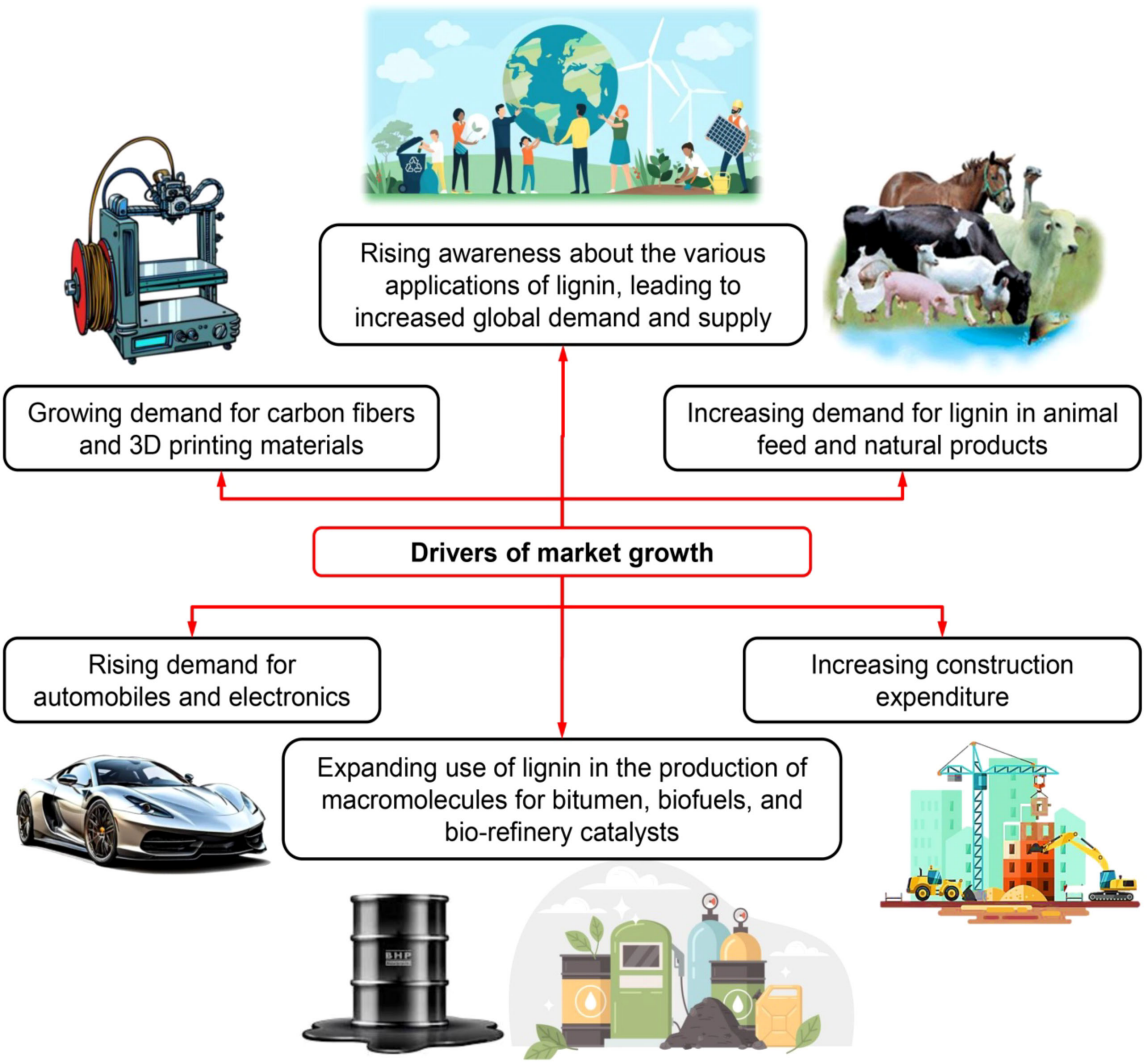
Key drivers of global lignin market growth across major application sectors.

From a market perspective, global demand for lignin and lignin-derived products is projected to grow substantially over the coming decade. The lignin market was valued at approximately USD 1.08 billion in 2023 and is expected to reach USD 1.98 billion by 2034, corresponding to a compound annual growth rate (CAGR) of approximately 4.5% (Mahbubul and Himan, 2023). Broader market analyses estimate the lignin and lignin-based products sector at USD 9.95 billion in 2022, with projections reaching USD 14.19 billion by 2029 (CAGR ≈ 5.2%) (Argyropoulos et al., 2023). This expansion is driven by multiple converging demand-side forces across industrial sectors, including construction, transportation, additive manufacturing, energy systems, agriculture, and bio-refinery operations. As illustrated in **Figure 7**, rising awareness of lignin’s multifunctionality—together with increasing demand for carbon fibers and 3D-printing materials, construction inputs, automotive and electronic components, animal feed additives, and bio-refinery intermediates—is accelerating both global lignin demand and supply. These interconnected drivers indicate that lignin market growth is not confined to a single application sector, but rather emerges from cross-sectoral adoption and diversification, reinforcing lignin’s role as a strategic renewable feedstock within the expanding bio-based economy. However, realizing this growth will require overcoming barriers related to variable lignin quality, capital-intensive processing infrastructure, regulatory complexity, and uneven market awareness, particularly in developing regions (Wenger et al., 2020).

Overall, lignin valorization offers a multifaceted pathway toward environmental sustainability, economic diversification, and technological innovation. By transforming an underutilized byproduct into a renewable feedstock for high-value applications, lignin can contribute meaningfully to emissions reduction, circular production systems, and green market development. As both scientific understanding and industrial capabilities continue to mature, lignin is positioned to become a foundational pillar of the emerging bioeconomy.

### 6.1 Life-cycle assessment evidence for lignin valorization pathways

LCA is essential for evaluating lignin valorization because claims of “renewability” alone do not guarantee lower impacts once separation, upgrading, and end-of-life are considered. A critical review of dozens of peer-reviewed LCAs covering lignin and lignin-derived products shows that the direction and magnitude of environmental benefits depend strongly on methodological choices (system boundaries, allocation, energy mix, and substitution assumptions) and on where energy- and solvent-intensive steps occur in the value chain (Moretti et al., 2021). In many LCAs, the most influential contributors are upstream fractionation and solvent recovery (where applicable), drying and purification to meet specifications for materials applications, and high-temperature processing steps in carbon materials routes (Moretti et al., 2021). The evidence therefore supports a conditional conclusion: lignin valorization can lower climate impacts compared to fossil-based alternatives, but only when processing energy is low-carbon and when separations and chemical inputs are efficiently recycled at high yield (Kylili et al., 2023).

Published case studies illustrate both opportunities and constraints. For lignin-to-carbon-fiber pathways, process-level LCAs show that climate impacts are driven by precursor preparation, stabilization, and carbonization energy demand, and that “advanced” process integration can reduce greenhouse gas emissions compared with conventional routes (Kawajiri and Sakamoto, 2022). For lignin-based resins and polymer intermediates, recent LCA modeling indicates potentially large impact reductions when lignin displaces fossil aromatics in phenolic resin systems; however, these results depend on assumptions about lignin sourcing, purification intensity, and what incumbent product is displaced (Miao et al., 2024). Finally, for integrated biorefinery routes that depolymerize lignin to chemicals, combined techno-economic analysis (TEA)–LCA frameworks demonstrate that solvent/catalyst recovery, hydrogen supply, and heat integration can dominate both environmental and economic outcomes (Bartling et al., 2021). These studies collectively support the need to evaluate lignin valorization in “whole-system” terms rather than judging sustainability solely from the feedstock origin.

### 6.2 The “zero-burden” assumption for waste lignin and why it can mislead

A frequent simplifying approach in lignin LCAs is to assign lignin a “zero burden” when it is treated as a waste or low-value byproduct, implying that lignin enters the valorization system without upstream environmental impacts. This assumption can be useful for exploring best-case scenarios, but it can substantially overstate benefits when lignin requires additional recovery operations (e.g., extraction from black liquor, precipitation, filtration, washing, drying) and when diverting lignin changes mill energy balances. A dedicated methodological study on allocation in lignin LCA demonstrates that results can change markedly depending on allocation choice (mass-, energy-, or economic allocation and/or system expansion) and recommends sensitivity analysis because lignin is inherently a co-product of multi-output systems (Hermansson et al., 2020). The same work emphasizes that many published lignin substitution case studies omit realistic post-treatment requirements (e.g., drying and purification) that would be needed for materials applications, which can bias impacts downward.

Consequential LCAs further show why the zero-burden framing may be inappropriate when lignin is currently used for energy recovery at the mill. A consequential assessment of kraft lignin recovery highlights that using lignin as an energy feedstock can be environmentally advantageous within the mill context, and that diverting lignin can shift burdens to replacement fuels and altered chemical recovery operations (Marson et al., 2023). For this reason, sustainability claims should explicitly state the allocation approach used, justify it based on the goal and scope, and include a sensitivity case where lignin carries a share of upstream impacts or where the displaced energy function is modeled explicitly (Hermansson et al., 2020).

### 6.3 Processing energy and chemical inputs versus the incumbent pathway of incineration for energy recovery

A critical sustainability analysis must compare lignin valorization not only against fossil-based alternatives, but also against the incumbent baseline in many industrial settings: lignin combustion for process heat and power and its role in chemical recovery cycles. Kraft lignin is commonly burned in recovery boilers, supporting the mill’s energy self-sufficiency and chemical recovery, and any diversion of lignin to materials must therefore be evaluated against the counterfactual of lost on-site energy and potentially altered recovery operations (Argyropoulos et al., 2023). In systems where lignin combustion displaces fossil fuels on-site, diverting lignin to a material pathway may require replacement energy, and the climate benefit of the material product must be large enough to offset this substitution effect (Marson et al., 2023).

Across published LCAs, a consistent pattern emerges: the “reactor” step is not always the dominant driver; instead, separations and thermal post-treatment can dominate total impacts (Moretti et al., 2021). This is especially relevant for high-purity lignin streams and performance-critical materials, where drying, solvent recovery, and purification can be substantial contributors. For carbon materials, energy-intensive stabilization and carbonization can be the main drivers, meaning that the electricity and heat mix strongly controls whether lignin-based fibers deliver meaningful greenhouse gas reductions relative to fossil-based fibers (Kawajiri and Sakamoto, 2022). Therefore, sustainability conclusions should be framed conditionally: lignin valorization is most defensible environmentally when the pathway (i) displaces carbon-intensive incumbents (fossil aromatics or high-impact materials), (ii) uses low-carbon energy, and (iii) minimizes solvent and chemical inputs through robust recycling and heat integration (Bartling et al., 2021).

### 6.4 Green premium and economic viability relative to incineration and fossil-based alternatives

Even when environmental performance is favorable, lignin valorization must overcome the “green premium,” defined as the additional cost required to deliver lower-carbon alternatives that match incumbent product performance. Integrated hotspot and sustainability-lever analyses of lignin valorization pathways show that economic feasibility frequently hinges on scale, solvent/catalyst recovery efficiency, energy integration, and product price sensitivity, rather than on lignin feedstock cost alone (Lettner et al., 2018). For emerging lignin conversion platforms, combined TEA–LCA studies indicate that hydrogen supply, catalyst lifetime, solvent recycling, and separation energy can simultaneously drive both operating cost and environmental impact, making process intensification and recycling central to reducing the green premium (Bartling et al., 2021).

Economic viability must also be evaluated against the opportunity cost of incineration, where lignin provides an established energy and recovery function (Argyropoulos et al., 2023). In many cases, near-term economically viable strategies are those that use lignin in partial substitution roles with minimal additional processing, such as replacing a portion of fossil aromatics in resins or serving as a functional additive, rather than routes requiring deep purification and multiple chemical transformations (Moretti et al., 2021). Recent TEA–LCA case studies for lignin-derived resins and polymer intermediates illustrate this point by showing that cost competitiveness improves when lignin can be used at meaningful substitution levels without extensive upgrading, while environmental gains are preserved through efficient processing and low-carbon energy (Staccioli et al., 2024). To synthesize the diverse life-cycle assessment outcomes reported for lignin valorization, **Table 6** summarizes representative LCA findings across major lignin utilization pathways, explicitly indicating allocation assumptions, dominant environmental hotspots, and the comparison baseline used in each study. Overall, the published evidence supports a realistic commercialization outlook: lignin valorization can be both sustainable and economically viable, but only when pathways are selected for high displacement value, integrated with existing infrastructure, and engineered to minimize energy and chemical intensity; otherwise, incineration for energy recovery remains the more robust baseline (Marson et al., 2023).

**Table 6:** Life-cycle assessment findings for major lignin valorization pathways.

Lignin valorization pathway	Reference product/baseline	Allocation approach used	Main environmental hotspot identified	Key sustainability conclusion	References
Lignin-derived carbon fibers	PAN-based carbon fibers; lignin incineration for energy	System expansion and economic allocation	Stabilization and carbonization energy demand; electricity mix	Climate benefits achievable only with low-carbon energy and efficient heat integration; not universally superior to incineration	Janssen et al. (2019); Kawajiri and Sakamoto (2022)
Lignin-substituted phenolic resins	Fossil phenol–formaldehyde resins	Mass and economic allocation	Lignin purification and resin processing	Partial phenol substitution reduces GHG emissions; benefits depend on lignin purity and substitution level	Lettner et al. (2018); Moretti et al. (2021)
Lignin-based polyols/PU foams	Fossil-based polyols	Economic allocation	Solvent use and chemical modification steps	Environmental benefits possible but sensitive to solvent recovery and functionalization route	Staccioli et al. (2024)
Catalytic depolymerization (RCF, aromatics)	Fossil aromatics and fuels	System expansion (displacement of fossil chemicals)	Hydrogen supply, catalyst production, solvent recovery	Sustainability strongly pathway-dependent; high impact if solvent/catalyst recycling is inefficient	Bartling et al. (2021)
Integrated kraft lignin recovery	On-site lignin incineration for heat and power	Consequential LCA	Loss of energy recovery and altered mill energy balance	Diverting lignin can increase impacts unless displaced products have high carbon intensity	Marson et al. (2023)
Mixed lignin-based products	Fossil-based materials and chemicals	Variable (mass, energy, economic)	Allocation choice and system boundaries	Reported benefits vary widely; allocation assumptions critically influence outcomes	Hermansson et al. (2020); Kylili et al. (2023)

PAN, polyacrylonitrile; PU, polyurethane; RCF, reductive catalytic fractionation; LCA, life-cycle assessment; GHG, greenhouse gas.

## 7 Challenges, future directions, and global sustainability prospects of lignin utilization

Lignin valorization stands at a critical intersection of plant biology, materials science, and industrial sustainability. While substantial progress has been achieved in lignin extraction, modification, and application development, the transition from laboratory-scale demonstrations to economically robust, large-scale deployment remains constrained by interrelated structural, technological, and systemic challenges. Addressing these limitations is essential for positioning lignin not merely as a renewable substitute, but as a strategically differentiated feedstock capable of underpinning next-generation circular bioeconomy systems.

### 7.1 Structural heterogeneity as a central scientific and technological barrier

The most persistent challenge in lignin utilization arises from its intrinsic structural heterogeneity. Lignin is not a single polymer but a population of macromolecules with variable monomer composition (H/G/S ratios), linkage distributions, molecular weights, and functional group densities that depend on plant species, tissue type, growth conditions, and extraction history. This variability directly undermines reproducibility, reactivity control, and predictable structure–property relationships, complicating both chemical upgrading and materials engineering (Vishtal and Kraslawski, 2011). Even within a single technical lignin stream, batch-to-batch variation can be substantial, limiting standardization and quality assurance at industrial scale.

Conventional pulping and extraction processes further exacerbate this challenge by introducing process-induced modifications, including condensation reactions, sulfur incorporation, ash contamination, and carbohydrate residues. These changes alter lignin solubility, thermal behavior, and functional group accessibility, reducing compatibility with polymer matrices and catalytic systems (Cline and Smith, 2017). Consequently, lignin heterogeneity must be viewed not as a secondary inconvenience, but as a primary design constraint that shapes every downstream valorization pathway. Future progress therefore depends on quality-engineered lignin streams, rather than bulk lignin recovery alone. Advances in fractionation, molecular-weight control, and selective extraction—such as DES-based systems, hydrotropic extraction, and post-isolation solvent fractionation—represent essential enabling technologies for reducing chemical diversity and improving reproducibility (Singh and Ahn, 2025). Without such upstream control, many advanced lignin-based applications will remain confined to niche or pilot-scale demonstrations.

### 7.2 Process compatibility and the need for lignin-specific conversion paradigms

A second major challenge lies in the fundamental mismatch between lignin chemistry and conversion technologies historically optimized for petroleum-derived feedstocks. Most industrial polymerization, catalytic, and refining processes were designed around relatively uniform hydrocarbon substrates, whereas lignin presents a multifunctional, oxygen-rich, and irregular aromatic network (Lizundia et al., 2021). As a result, direct transfer of petrochemical processing logic to lignin often leads to low selectivity, poor yields, and high energy demand. In materials applications, this mismatch manifests as poor interfacial adhesion, phase separation, brittleness, and narrow processing windows when lignin is blended into conventional polymers. In chemical depolymerization routes, uncontrolled recondensation, catalyst deactivation, and complex product mixtures remain persistent issues (Vanarse et al., 2025). Overcoming these challenges requires lignin-first or lignin-specific process design, rather than incremental adaptation of fossil-based technologies. Encouragingly, recent advances in catalytic fractionation, enzyme-mediated functionalization, and solvent-assisted stabilization demonstrate that lignin’s reactivity can be harnessed selectively when chemistry is designed around its molecular features rather than against them. However, such approaches often involve higher capital intensity, catalyst sophistication, or solvent recovery demands, reinforcing the need for integrated techno-economic and life-cycle optimization rather than isolated process innovation.

### 7.3 Economic constraints and the “green premium” challenge

Even where technical feasibility has been demonstrated, economic competitiveness remains a decisive barrier. Lignin valorization pathways frequently suffer from high processing costs associated with extraction, purification, solvent recovery, catalyst use, and energy-intensive thermal steps (Jiju et al., 2025). These costs are particularly challenging when lignin-derived products compete directly with mature fossil-based incumbents that benefit from decades of optimization, scale, and infrastructure. A critical but often underappreciated economic factor is the opportunity cost of lignin incineration. In many pulp mills and biorefineries, lignin currently provides low-cost, on-site energy and supports chemical recovery cycles (Agnihotri and Horváth, 2024). Diverting lignin to materials or chemicals therefore requires not only covering additional processing costs, but also compensating for lost energy functions. This reality fundamentally reshapes economic comparisons and reinforces the importance of evaluating lignin valorization against realistic counterfactual baselines rather than fossil-only benchmarks. Near-term economically viable strategies are therefore most likely to involve partial substitution and functional upgrading, such as replacing a fraction of fossil aromatics in resins, serving as performance-enhancing additives, or targeting applications where sustainability premiums, regulatory pressure, or functional differentiation justify higher costs (Musa et al., 2024). Deep depolymerization routes to commodity aromatics, by contrast, remain economically fragile unless supported by breakthroughs in catalyst lifetime, hydrogen sourcing, and process integration.

### 7.4 Sustainability assessment beyond “renewable” narratives

From an environmental perspective, lignin’s renewable origin does not automatically guarantee superior sustainability outcomes. LCA studies consistently show that environmental benefits depend strongly on system boundaries, allocation assumptions, energy mix, solvent and catalyst recovery efficiency, and the choice of displaced incumbent products (Artz et al., 2018). The widespread use of the “zero-burden” assumption—treating lignin as impact-free because it is a byproduct—can significantly overestimate climate benefits when additional recovery, drying, and purification steps are required. Moreover, comparisons must explicitly account for the incumbent role of lignin combustion for energy recovery. In systems where lignin displaces fossil fuels on-site, diverting it to materials or chemicals can increase emissions unless the valorized product delivers sufficiently high displacement value elsewhere (Dey et al., 2025). Consequently, the most defensible sustainability claims arise from integrated system-level analyses that evaluate lignin valorization alongside energy substitution, heat integration, and infrastructure context. Future research must therefore embed sustainability assessment directly into process and product design, using combined TEA–LCA frameworks to identify hotspots, guide technology selection, and avoid burden shifting. Pathways that minimize separation intensity, rely on low-carbon energy, and achieve high material substitution efficiency consistently emerge as the most robust options.

### 7.5 Future directions for aligning biology, chemistry, and systems design

Looking forward, the most transformative opportunities in lignin utilization lie at the intersection of plant biotechnology, molecular engineering, and process integration. Advances in lignin biosynthesis engineering—including altered S/G ratios, increased β–O–4′ content, and incorporation of cleavable or “zip-lignin” motifs—demonstrate that lignin structure itself can be redesigned to improve extractability and convertibility without compromising plant performance (Boarino and Klok, 2023). Such approaches shift part of the valorization challenge upstream, reducing downstream processing severity and cost. In parallel, continued development of selective extraction, fractionation, and modification strategies will be essential for producing application-ready lignin streams tailored to specific value chains (Wu et al., 2025b). Rather than pursuing a single dominant application, future lignin valorization is likely to follow a portfolio model, where different lignin fractions are directed toward materials, additives, carbon products, or chemical intermediates based on quality and market demand. Interdisciplinary collaboration will be critical in this context. Bridging plant science, catalysis, polymer engineering, sustainability assessment, and industrial design requires coordinated efforts across academia, industry, and policy frameworks. Regulatory incentives, carbon pricing mechanisms, and green procurement policies can further accelerate adoption by internalizing environmental benefits that are not fully captured by market prices (Samuel, 2025).

### 7.6 Global sustainability prospects and concluding perspective

In a global context, lignin represents more than a technical feedstock; it embodies a strategic opportunity to decouple aromatic materials production from fossil resources while strengthening rural bioeconomies and reducing industrial carbon intensity (Ekielski and Mishra, 2020). Its successful deployment can contribute directly to multiple United Nations Sustainable Development Goals, including climate action, responsible consumption and production, and sustainable industrialization. Ultimately, the future of lignin utilization will not be defined by the elimination of its complexity, but by the ability to harness and manage that complexity intelligently (Bin Abu Sofian et al., 2024). When structural heterogeneity is controlled rather than ignored, when processing is designed around lignin’s chemistry rather than forced into fossil paradigms, and when sustainability is evaluated at the system level rather than assumed, lignin can transition from an underutilized byproduct to a cornerstone material of the global bioeconomy. In summary, lignin’s path toward large-scale impact depends on harmonizing scientific innovation, economic realism, and sustainability principles. Continued advances in biology, chemistry, and systems engineering—supported by informed policy and market mechanisms—will determine whether lignin fulfills its promise as a renewable platform for materials, chemicals, and climate-resilient industrial systems.

## 8 Conclusion

Lignin has emerged from a largely undervalued industrial byproduct to a strategically important renewable resource at the intersection of plant biology, materials science, and circular bioeconomy development. As the only large-volume renewable source of aromatic carbon, lignin offers distinctive opportunities to displace fossil-derived chemicals and materials while contributing to decarbonization and resource efficiency. This review highlights how advances in lignin extraction, fractionation, and modification have expanded the range of feasible valorization pathways toward functional materials and renewable aromatic chemicals. A key conclusion is that lignin heterogeneity represents both the central technical challenge and a critical design parameter. While structural variability complicates processing and reproducibility, it can be effectively managed through targeted fractionation, controlled modification, and application-specific design. In this context, green extraction strategies—particularly deep eutectic solvent and hydrotropic systems—demonstrate that lignin isolation can function as a quality-engineering step that directly influences downstream performance. Across material applications, quantitative benchmarking shows that lignin-derived systems are most competitive when deployed as functional and sustainability-driven alternatives rather than direct drop-in replacements. Promising opportunities are evident in carbon fibers for non-aerospace uses, as well as in nanomaterials, adhesives, cementitious systems, additive manufacturing, and bioplastics, where lignin’s aromatic structure and surface functionality enable targeted performance advantages. From a systems perspective, lignin valorization must be assessed against realistic baselines, particularly its conventional use in energy recovery. Life-cycle evidence indicates that environmental benefits are conditional and depend on allocation choices, energy sources, and process integration. Looking ahead, aligning plant-derived structural diversity with industrial convertibility—supported by advances in biosynthesis engineering, process integration, and policy frameworks—will be essential. Rather than a single dominant application, lignin’s future impact will arise from a portfolio of application-targeted solutions that leverage its unique molecular features within integrated biorefinery systems.
